# Global burden of esophagus cancer, stomach cancer and colon and rectal cancer from 1990 to 2050: A cross-sectional study

**DOI:** 10.1097/MD.0000000000050376

**Published:** 2026-08-21

**Authors:** Rao Wang, Wei-Guang Liu

**Affiliations:** a Thyroid and Breast Surgery Department, Yangzhou Maternal and Child Health Hospital Affiliated to Yangzhou University, Yangzhou, Jiangsu, China.

**Keywords:** Bayesian age-period-cohort model, estimated annual percentage change, gastrointestinal tract cancer, Global Burden of Disease, socio-demographic index

## Abstract

Esophageal cancer, stomach cancer, and colon and rectal cancer (CRC), as the main components of gastrointestinal tract cancer (GTC), represent significant obstacles to reducing the global cancer burden. Therefore, obtaining an epidemiological characteristic of the 3 major GTCs is highly important. This study aims to analyze and forecast the changing trends in the burden of the 3 major GTCs, offering a certain theoretical foundation for cancer public health policymakers. The data utilized in this study were all sourced from the Global Burden of Disease study 2021. Specifically, the case numbers and age-standardized rate (ASR) for prevalence, death, and disability-adjusted life years of esophageal cancer, stomach cancer, and CRC were included. Additionally, the estimated annual percentage change of the 3 major GTCs was computed for the period spanning from 1990 to 2021 across 5 distinct socio-demographic index and 21 Global Burden of Disease regions. Moreover, a Bayesian age-period-cohort model was constructed to project the disease burden of the 3 major GTCs from 2022 to 2050. From 1990 to 2021, the number of cases of the 3 major GTCs showed an overall increasing trend. Regarding ASRs, with the exception of the increasing age-standardized prevalence rate of CRC, the ASRs of the remaining GTCs demonstrated a downward trend. The disease burden of the 3 GTCs was higher among males compared to females, predominantly affecting individuals aged 50 and above. The regions with notable ASRs of the 3 major GTCs were East Asia and high-income Asia. The prediction outcomes also suggested that, apart from the age-standardized prevalence rate of CRC, the burden of the 3 major GTCs might display a decreasing trend in the next 29 years. Although the ASRs of the 3 primary types of GTC exhibit a downward trend, the case counts of these types still maintain a relatively high baseline level. Consequently, conducting GTC screening in specific regions, developing relevant health policies and popularizing disease-related knowledge continue to hold critical significance.

## 1. Introduction

Gastrointestinal cancer (GC) ranks among the most common cancers worldwide, and gastrointestinal tract cancer (GTC) constitutes the major component of GC.^[1]^ Among GTC, esophagus cancer (EC), stomach cancer (SC), and colon and rectal cancer (CRC) are the most common. Drawing upon the Global Cancer Observatory (GLOBOCAN) dataset, scholars reported that in 2020, there were 0.60 million incident cases and 0.54 million deaths cases of EC globally; 1.09 million incident cases and 0.77 million death cases of SC; and 1.9 million incident cases and 0.935 million deaths cases of CRC.^[2]^ Consequently, the epidemiological situation of GTC remains grave.

Presently, scholars have employed the Surveillance, Epidemiology, and End Results database to analyze changing patterns in the age-standardized incidence rate (ASIR) for EC in the United States from 1975 to 2018. The findings indicated that the ASIR of EC rose from 4.14 per 100,000 in 1975 to 4.18 per 100,000 in 2018, demonstrating a gradual upward trend.^[3]^ However, the data utilized in the study were predominantly sourced from the United States. The prevalence and death epidemiological patterns of GTC exhibit substantial variations across different regions. For instance, esophageal adenocarcinoma constitutes the predominant subtype of EC in Western countries, whereas esophageal squamous cell carcinoma, accounting for 85% of cases, is more prevalent in Asia and South Africa.^[4]^ In addition, *Helicobacter pylori (H pylori*) infection is recognized as the greatest risk factor for SC in Asian populations, while long-term heavy alcohol consumption is the most critical risk factor for SC in Latin American populations.^[5]^ In addition, the high-risk factors for CRC include smoking and consumption of red meat and processed meat.^[6]^ The burden of CRC varies between regions with different income levels due to differences in smoking rates and red meat consumption.^[6]^ This circumstance has undoubtedly underscored the significance of analyzing the epidemiological status of the 3 major GTCs. Remarkably, despite the fact that scholars have evaluated the ASIR and age-standardized death rate (ASDR) of GC based on the Global Burden of Disease (GBD) 2021.^[1]^ However, this study did not encompass an analysis of the prevalence and disability-adjusted life years (DALYs) of the 3 major GTCs. Consequently, gaining comprehensive insight into the global epidemiological landscape of these 3 primary GTCs and implementing effective measures to mitigate their disease burden is imperative.

Accordingly, this study leverages the GBD 2021 database to conduct an analysis and forecasting of temporal trends in the burden of prevalence, death, and DALYs associated with the 3 major GTCs on a global scale. The findings derived from this investigation are anticipated to furnish critical theoretical underpinnings for informing public health decision-making and guiding strategic measures aimed at mitigating the disease burden of these 3 predominant GTCs worldwide.

## 2. Methods

The data utilized in this study were obtained from the GBD 2021 database (https://vizhub.healthdata.org/gbd-results/). Launched by the Institute for Health Metrics and Evaluation at the University of Washington, the GBD 2021 represents a global research endeavor that monitors the epidemiological trends of 371 diseases and injuries across 204 countries.^[7]^ The GBD 2021 incorporates data from 100,983 global sources. Nonfatal health-related data are obtained from the literature, epidemiological questionnaires, disease surveillance systems, health registration systems, and clinical information databases. Meanwhile, fatal health-related data are derived from population dynamics registration systems, standardized autopsy reports, specialized epidemiological surveys, national census data, and professional medical records.^[7]^ This study incorporated the prevalence, death, and DALYs numbers of cases, along with the age-standardized rate (ASR) of EC, SC, and CRC stratified by sex and region.

This research utilizes ASR to explain the variations in age distribution and population size, thereby facilitating effective cross-regional and temporal comparisons. Both the number of cases and ASR are quantified with 95% uncertainty interval (UI) to quantify the uncertainties in estimation.^[7]^ To evaluate the time-varying trends of the 3 primary GTCs, we computed the estimated annual percentage change (EAPC), encompassing age-standardized prevalence rate (ASPR), ASDR, and age-standardized DALY rate (ASDALYR).^[8]^ The EAPC is computed by utilizing the time-series data of the ASR. By conducting a log-linear regression, where the natural logarithm of the ASR serves as the dependent variable and the year functions as the independent variable, the regression equation is derived. Subsequently, the coefficients are transformed into percentages (formula: EAPC = (*e*^β^ − 1)^*^100%), which reflects the long-term trends of the burden. A positive EAPC value with a 95% confidence interval (CI) above 0 implies an upward trend in the ASR, whereas a negative value indicates a downward trend.^[9]^

Furthermore, this study encompasses 204 countries and regions, which were stratified into 21 GBD regions based on geographical proximity and categorized into 5 groups according to quintiles of the socio-demographic index (SDI): low (0–0.45), low–middle (0.45–0.61), middle (0.61–0.69), high–middle (0.69–0.81), and high (0.81–1).^[7,10]^ Higher SDI values are associated with more favorable socioeconomic circumstances and superior health outcomes.

Furthermore, leveraging global age-specific burden data for the 3 major GTCs spanning 1990 to 2021, this study utilized Bayesian age-period-cohort (BAPC) model to project the disease burden of these cancers from 2022 to 2050.^[11]^ The BAPC model is predicated on the assumption that age, period, and cohort effects exhibit temporal proximity. Within the model, Bayesian inference employs a 2nd-order random walk to smooth the priors of these 3 parameters and estimate posterior rates. Regarding prior selection, we adopted random walk of order 2 (RW2) rather than RW1 because it offers a better balance between model flexibility and smoothness, making it particularly suitable for epidemiological data expected to exhibit nonlinear long-term trends.^[12,13]^ The precision parameters employ a Gamma distribution with weak informativeness (shape = 1, rate = 0.0005), which is the standard recommended configuration for the BAPC model.^[12]^ It is noteworthy that the RW2 prior in the BAPC model is essentially a nonstationary process, which does not assume that period and cohort effects are stationary but rather achieves smooth estimation by penalizing drastic changes between adjacent time points. This approach has proven advantageous in capturing nonlinear trends in epidemiological data.^[12,14]^ The selection of the BAPC model was further supported by prior comparative studies. Huang et al systematically evaluated 5 prediction models (classical APC Poisson model, BAPC model, Poisson regression, negative binomial regression, and generalized additive model) using GBD data, demonstrating that the BAPC model exhibited the lowest overall mean absolute percentage error and provided well-calibrated projections with appropriately narrow uncertainty intervals.^[15]^ Liu et al further corroborated that the superior predictive performance of the BAPC model compared to Joinpoint regression, Poisson regression, and other prediction models has been previously verified.^[16]^ Furthermore, BAPC utilizes integrated nested Laplace approximation to approximate marginal posterior distributions, thereby circumventing the mixing and convergence issues inherent in traditional Bayesian methods reliant on Markov Chain Monte Carlo approaches.^[17,18]^ Finally, to address potential structural 0-value issues in low-burden regions, we employed a standard preprocessing method of adding a small constant (0.5) to 0-value cells during data preparation.^[19]^ And the Poisson likelihood framework inherent in the BAPC model inherently handles 0-count scenarios.^[12]^ This study also analyzed the attributable risk factors for 3 major GTCs. All risk factors classified as GBD risk factor level 3 were included. The population attributable fraction (PAF) for these 3 GTCs was quantified. PAF represents the proportion of the outcome that can be reduced when current exposure is lowered to the theoretical minimum risk exposure level.^[20]^

To elucidate the independent contributions of demographic and epidemiological transitions to the changing burden of EC, SC, and CRC, we employed the Das Gupta decomposition method.^[21]^ This approach algebraically partitions the net change in absolute numbers of prevalent cases, deaths, and DALYs between 1990 and 2021 into 3 additive components: population growth, reflecting changes in total population size; population aging, reflecting shifts in population age structure; and epidemiological changes, reflecting alterations in age-specific disease rates independent of demographic shifts, encompassing changes in risk factor exposure, screening practices, treatment advances, and disease management.^[22]^

All aforementioned statistical analyses were performed using R (version 4.3.2; R Foundation for Statistical Computing).

This study employed the International Classification of Diseases coding system to standardize all disease terminology, aiming to ensure data accuracy and enhance the comparability of research findings. GC is classified under International Classification of Diseases-10 codes C15-C26. Among these, the 3 major GTCs focused on in this study include EC (C15), SC (C16), and CRC (C18–C20). Furthermore, the implementation of this project strictly adhered to the guidelines outlined in the Guidelines for Accurate and Transparent Health Estimates Reporting.

## 3. Results

### 3.1. Esophagus cancer

**Figure 1.**
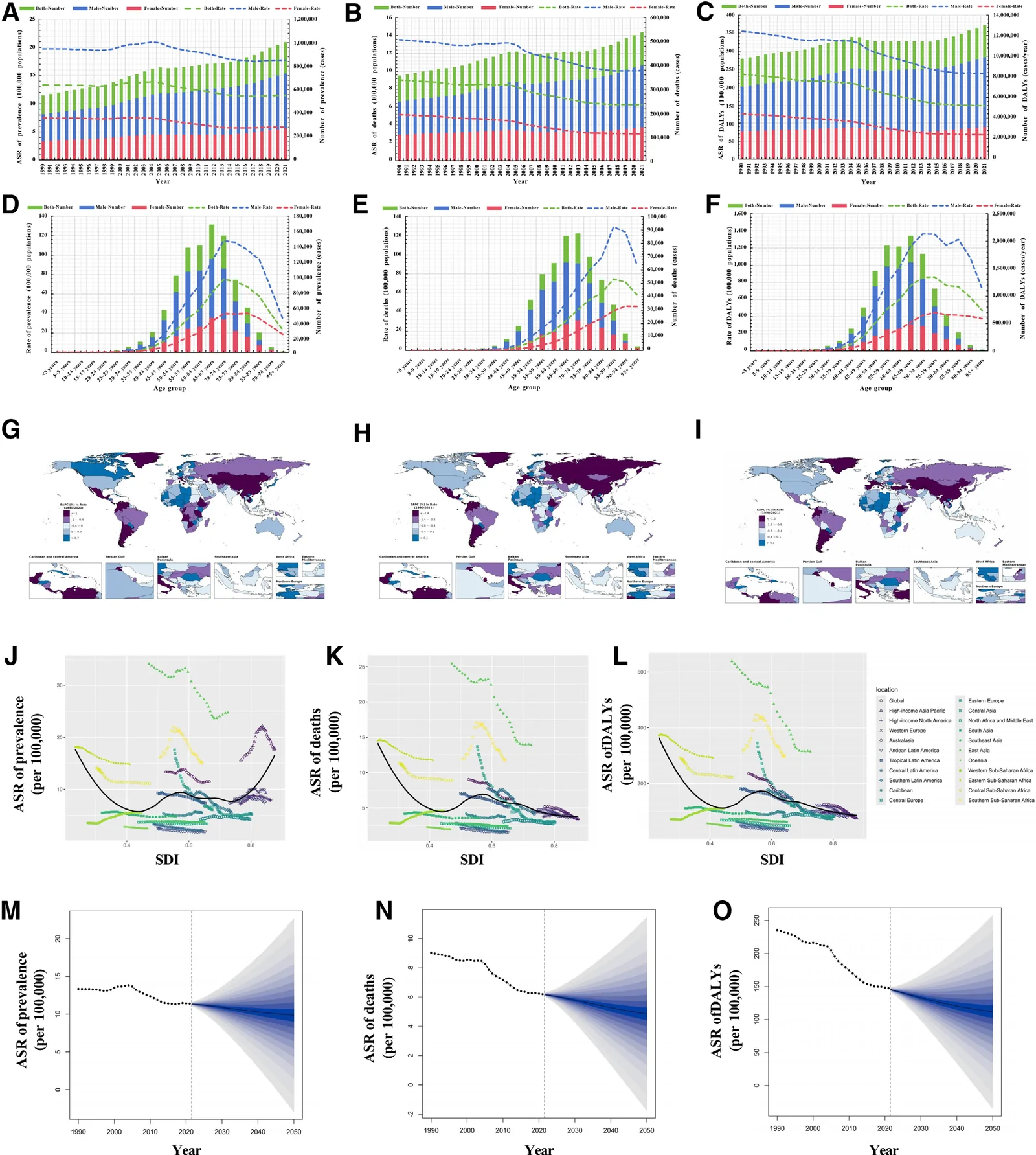
Trends in disease burden of EC (1990–2050). (A–C) Show prevalence, deaths, and DALYs burden trends for EC across different years and genders from 1990 to 2021; (D–F) present age-specific and gender-disaggregated trends for EC’s prevalence, deaths, and DALYs burden during the same period; (G–I) display global mapping of EC’s prevalence, deaths, and DALYs using the EAPC; (J–L) illustrate ASR of prevalence, deaths, and DALYs across 21 GBD regions with varying SDIs; and (M–O) present ASR of prevalence, deaths, and DALYs with projected 2050 projections from the BAPC model. ASR = age-standardized rate, BAPC = Bayesian age-period-cohort, DALYs = disability-adjusted life years, EAPC = estimated annual percentage change, EC = esophageal cancer, GBD = Global Burden of Disease, SDIs = socio-demographic indexes.

On a global scale, the number of prevalent cases escalated from 0.55 million cases in 1990 to 1.00 million cases in 2021, signifying an 82.08% elevation; the number of deaths ascended from 0.36 million cases to 0.54 million cases, registering a 51.18% growth; and the number of DALYs increased from 9.75 million cases to 13.00 million cases, indicating a 33.28% upsurge. Conversely, the ASPR, ASDR, and ASDALYR of EC demonstrated a downward trend during the period from 1990 to 2021. Notably, the ASDALYR decreased from 235.32 per 100,000 (95% UI: 210.52–258.68) to 148.56 per 100,000 (95% UI: 131.71–166.82), with an EAPC of −1.73 (95% CI: −1.87 to −1.59) (Table 1). In 2021, the global burden of EC was greater among males than females, with the most prominent disparity observed in the ASDALYR, which was 3.51 times higher in males (Fig. 1A–C). Moreover, the burden of EC exhibited a strong age-related dependence: beyond the age of 50 years, it increased sharply with advancing age (Fig. 1D–F).

**Table 1:** Trends in esophagus cancer burden across 5 SDI and 22 GBD regions.

Location	Prevalence					Deaths					DALYs				
	Number, 1990 (95% UI)	Number, 2021, (95% UI)	ASR, 1990 (95% UI)	ASR, 2021 (95% UI)	EAPC, 1990–2021 (95% CI)	Number, 1990 (95% UI)	Number, 2021 (95% UI)	ASR, 1990 (95% UI)	ASR, 2021 (95% UI)	EAPC, 1990–2021 (95% CI)	Number, 1990 (95% UI)	Number, 2021 (95% UI)	ASR, 1990 (95% UI)	ASR, 2021 (95% UI)	EAPC, 1990–2021 (95% CI)
High–middle SDI	168,398 (147,147–189,115)	304,466 (249,662–368,826)	16.34 (14.29–18.34)	15.27 (12.52–18.51)	−0.41 (−0.57 to −0.24)	114,332 (100,753–128,040)	162,430 (134,262–195,467)	11.52 (10.19–12.87)	8.13 (6.72–9.77)	−1.38 (−1.58 to −1.18)	3,116,679 (2,731,977–3,509,251)	3,834,291 (3,157,464–4,667,629)	303.10 (265.93–341.1)	192.56 (158.7–234.03)	−1.75 (−1.95 to −1.55)
High SDI	105,258 (101,728–107,564)	222,446 (208,686–231,816)	9.78 (9.47–9.99)	11.26 (10.62–11.71)	0.48 (0.19–0.78)	54,240 (51,962–55,653)	85,652 (79,160–89,950)	4.93 (4.73–5.06)	4.02 (3.75–4.2)	−0.78 (−0.89 to −0.67)	1,325,837 (1,286,516–1,357,512)	1,825,459 (1,719,026–1,902,714)	123.99 (120.47–126.95)	93.95 (89.28–97.92)	−1.01 (−1.14 to −0.89)
Low–middle SDI	37,992 (34,491–43,743)	78,269 (70,781–90,058)	5.68 (5.14–6.52)	5.10 (4.62–5.86)	−0.42 (−0.46 to −0.38)	26,141 (23,617–30,035)	53,724 (48,513–61,806)	4.36 (3.92–5.02)	3.79 (3.42–4.39)	−0.53 (−0.57 to −0.48)	763,111 (692,292–878,969)	1,491,634 (1,348,142–1,724,084)	113.84 (102.95–130.89)	97.10 (87.74–111.84)	−0.6 (−0.65 to −0.55)
Low SDI	22,368 (18,459–25,201)	41,837 (35,416–48,306)	9.03 (7.51–10.17)	7.53 (6.42–8.66)	−0.74 (−0.81 to −0.67)	15,828 (13,205–17,869)	28,924 (24,611–33,445)	7.15 (5.97–8.01)	5.89 (5.02–6.8)	−0.78 (−0.85 to −0.7)	460,138 (380,391–517,924)	830,121 (701,259–964,024)	185.38 (154.42–209.22)	148.67 (126.11–172.2)	−0.89 (−0.97 to −0.82)
Middle SDI	217,378 (181,667–251,978)	356,805 (297,605–425,220)	19.44 (16.25–22.47)	12.82 (10.72–15.25)	−1.63 (−1.77 to −1.48)	145,568 (123,678–168,238)	207,634 (174,863–246,498)	14.31 (12.19–16.45)	7.91 (6.65–9.34)	−2.18 (−2.34 to −2.02)	4,083,703 (3,438,091–4,731,710)	5,011,783 (4,233,898–5,964,294)	365.58 (309.42–422.25)	180.65 (153.17–214.63)	−2.57 (−2.73 to −2.41)
Andean Latin America	525 (461–599)	1083 (878–1346)	2.52 (2.22–2.87)	1.82 (1.48–2.26)	−1.12 (−1.25 to −0.99)	418 (369–476)	866 (712–1063)	2.18 (1.91–2.47)	1.51 (1.24–1.85)	−1.18 (−1.25 to −1.11)	10,238 (8990–11,648)	19,168 (15,515–23,634)	48.90 (42.88–55.55)	32.27 (26.17–39.76)	−1.43 (−1.55 to −1.31)
Australasia	1732 (1641–1828)	3949 (3579–4233)	7.37 (6.99–7.75)	7.62 (6.95–8.14)	0.16 (−0.01 to 0.32)	1021 (967–1081)	2050 (1849–2204)	4.34 (4.11–4.6)	3.68 (3.33–3.94)	−0.23 (−0.37 to −0.09)	23,048 (21,901–24,259)	41,017 (37,626–43,815)	98.88 (93.89–104.06)	80.20 (74.13–85.45)	−0.75 (−0.82 to −0.67)
Caribbean	1413 (1327–1502)	2959 (2596–3352)	5.38 (5.06–5.72)	5.47 (4.79–6.19)	0.36 (0.22–0.49)	1057 (998–1121)	1997 (1755–2254)	4.18 (3.95–4.43)	3.68 (3.24–4.16)	−0.61 (−0.77 to −0.46)	26,249 (24,499–28,020)	51,045 (44,533–58,194)	100.04 (93.47–106.74)	94.29 (82.3–107.49)	0.09 (−0.05 to 0.23)
Central Asia	8542 (8174–8914)	5217 (4631–5826)	17.58 (16.79–18.35)	6.02 (5.37–6.69)	−3.52 (−3.71 to −3.33)	6279 (5978–6568)	3735 (3342–4158)	13.68 (13–14.33)	4.74 (4.28–5.25)	−3.29 (−3.42 to −3.15)	167,513 (160,140–174,957)	99,947 (88,486–112,299)	344.55 (328.85–359.79)	115.48 (102.92–128.96)	−3.63 (−3.8 to −3.46)
Central Europe	6342 (6091–6617)	8628 (7899–9317)	4.21 (4.04–4.39)	4.26 (3.9–4.61)	−0.18 (−0.32 to −0.03)	4481 (4314–4659)	5926 (5441–6394)	3.02 (2.9–3.13)	2.76 (2.54–2.98)	−0.02 (−0.18 to 0.14)	122,471 (117,760–127,796)	146,485 (134,849–158,439)	81.56 (78.47–85.1)	73.05 (67.15–79.02)	−0.62 (−0.77 to −0.47)
Central Latin America	2861 (2787–2920)	5433 (4847–6120)	3.34 (3.23–3.41)	2.14 (1.91–2.41)	−1.54 (−1.62 to −1.46)	2180 (2101–2232)	4029 (3594–4524)	2.84 (2.72–2.91)	1.65 (1.47–1.85)	−1.92 (−1.98 to −1.87)	54,643 (53,217–55,742)	95,847 (85,462–107,643)	63.52 (61.67–64.93)	37.71 (33.67–42.31)	−1.8 (−1.89 to −1.72)
Central sub-Saharan Africa	3541 (2582–4510)	6879 (5006–8949)	14.11 (10.4–17.72)	11.13 (8.15–14.43)	−0.9 (−1 to −0.79)	2471 (1823–3120)	4653 (3398–6069)	11.36 (8.5–14.21)	8.89 (6.44–11.5)	−0.88 (−0.93 to −0.82)	73,191 (53,295–93,508)	137,615 (100,038–180,215)	291.65 (214.18–368.57)	221.54 (161.83–289.26)	−1.04 (−1.14 to −0.94)
East Asia	318,749 (264,546–371,429)	559,319 (449,552–685,965)	34.11 (28.32–39.61)	24.74 (19.92–30.25)	−1.31 (−1.51 to −1.12)	213,973 (179,118–247,919)	302,582 (243,363–368,743)	25.43 (21.28–29.31)	13.91 (11.23–16.84)	−1.86 (−2.03 to −1.68)	5,942,205 (4,927,391–6,910,479)	7,069,761 (5,660,281–8,736,103)	638.45 (532.3–740.63)	313.94 (252.18–387.12)	−2.62 (−2.85 to −2.4)
Eastern Europe	18,717 (18,330–19,117)	17,448 (15,737–18,955)	6.54 (6.4–6.68)	5.16 (4.65–5.61)	−0.93 (−1.2 to −0.66)	12,470 (12,181–12,701)	10,305 (9378–11,159)	4.41 (4.3–4.49)	2.94 (2.68–3.19)	−1.12 (−1.29 to −0.94)	340,939 (334,488–348,026)	274,133 (247,183–298,314)	119.37 (116.94–121.72)	81.25 (73.21–88.53)	−1.49 (−1.69 to −1.29)
Eastern sub-Saharan Africa	14,771 (11,992–17,002)	27,457 (22,777–33,147)	18.11 (14.85–20.85)	14.80 (12.35–17.81)	−0.82 (−0.88 to −0.76)	10,523 (8662–12,124)	19,000 (15,876–22,909)	14.52 (11.97–16.7)	11.74 (9.82–14.12)	−0.61 (−0.69 to −0.53)	304,871 (246,943–350,913)	545,442 (452,300–659,237)	373.02 (305.58–429.13)	292.22 (243.43–352.18)	−1 (−1.08 to −0.92)
Global	551,616 (493,219–604,999)	1,004,202 (888,169–1,120,957)	13.34 (11.94–14.61)	11.47 (10.15–12.8)	−0.64 (−0.79 to −0.5)	356,263 (319,363–390,154)	538,602 (475,944–603,406)	9.02 (8.11–9.87)	6.25 (5.53–7)	−1.11 (−1.22 to −0.99)	9,753,566 (8,719,319–10,739,561)	12,999,265 (11,522,861–14,605,268)	235.32 (210.52–258.68)	148.56 (131.71–166.82)	−1.73 (−1.87 to −1.59)
High-income Asia Pacific	32,285 (30,846–33,496)	76,564 (69,634–80,758)	15.59 (14.87–16.17)	17.79 (16.48–18.73)	0.44 (0.07–0.81)	10,304 (9798–10,770)	16,914 (15,031–17,974)	5.13 (4.87–5.37)	3.42 (3.1–3.62)	−1.23 (−1.3 to −1.15)	255,728 (244,998–268,388)	318,613 (290,566–336,610)	123.39 (118.12–129.46)	74.52 (69.25–78.49)	−1.8 (−1.94 to −1.66)
High-income North America	23,889 (22,968–24,380)	49,550 (47,126–51,314)	7.17 (6.93–7.31)	7.96 (7.6–8.23)	0.29 (0.08 to 0.5)	12,927 (12,268–13,264)	23,960 (22,394–24,952)	3.73 (3.55–3.82)	3.62 (3.4–3.76)	0.08 (−0.03 to 0.19)	312,474 (301,435–318,672)	535,321 (510,487–552,767)	94.58 (91.53–96.39)	86.13 (82.45–88.83)	−0.39 (−0.51 to −0.26)
North Africa and Middle East	6676 (5262–7684)	15,908 (13,494–17,858)	3.69 (2.94–4.23)	3.36 (2.88–3.78)	−0.4 (−0.49 to −0.31)	4466 (3585–5136)	8846 (7500–9959)	2.79 (2.26–3.19)	2.11 (1.81–2.36)	−0.83 (−0.88 to −0.79)	124,886 (97,851–144,297)	230,171 (191,966–262,991)	68.85 (54.78–79.48)	48.01 (40.39–54.41)	−1.28 (−1.34 to −1.23)
Oceania	91 (68–124)	203 (159–257)	2.80 (2.14–3.71)	2.45 (1.94–3.08)	−0.44 (−0.47 to −0.41)	61 (47–81)	134 (106–170)	2.29 (1.8–2.95)	1.95 (1.54–2.45)	−0.49 (−0.51 to −0.46)	1825 (1366–2436)	3906 (3056–5016)	56.05 (42.94–73.41)	47.10 (37.29–59.95)	−0.56 (−0.59 to −0.54)
South Asia	35,434 (31,519–42,952)	75,493 (66,650–89,744)	5.49 (4.88–6.64)	4.80 (4.24–5.72)	−0.67 (−0.77 to −0.57)	23,827 (21,092–28,894)	51,542 (45,651–61,688)	4.17 (3.66–5.06)	3.54 (3.12–4.26)	−0.51 (−0.6 to −0.41)	714,773 (635,598–868,530)	1,434,797 (1,268,757–1,704,574)	110.56 (97.8–134.35)	91.08 (80.54–108.48)	−0.86 (−0.96 to −0.76)
Southeast Asia	10,666 (8935–12,540)	26,443 (22,689–30,604)	3.81 (3.21–4.46)	3.74 (3.22–4.31)	−0.1 (−0.14 to −0.06)	7108 (5964–8306)	15,830 (13,725–18,154)	2.83 (2.39–3.3)	2.44 (2.13–2.78)	−0.48 (−0.51 to −0.44)	206,568 (172,765–242,795)	437,488 (374,888–504,001)	73.65 (61.86–86.17)	61.71 (53.19–70.79)	−0.63 (−0.68 to −0.58)
Southern Latin America	4436 (4249–4619)	4711 (4404–5042)	9.53 (9.12–9.93)	5.45 (5.11–5.83)	−1.8 (−2.01 to −1.59)	3500 (3342–3656)	3627 (3346–3879)	7.75 (7.38–8.11)	4.07 (3.77–4.35)	−1.74 (−1.89 to −1.59)	82,332 (78,920–85,738)	76,790 (72,068–82,063)	177.31 (169.83–184.56)	88.92 (83.56–94.99)	−2.24 (−2.44 to −2.03)
Southern sub-Saharan Africa	4783 (4298–5493)	9399 (8582–10,316)	16.16 (14.44–18.62)	15.08 (13.76–16.5)	−0.6 (−1.01 to −0.18)	3189 (2852–3705)	6602 (6026–7225)	11.89 (10.59–13.9)	11.69 (10.68–12.72)	0.09 (−0.23 to 0.41)	94,527 (85,131–108,239)	185,849 (169,360–204,414)	318.41 (285.54–365.34)	297.67 (271.83–326.47)	−0.6 (−1.08 to −0.13)
Tropical Latin America	9102 (8797–9348)	19,327 (18,382–20,085)	9.30 (8.94–9.56)	7.33 (6.96–7.62)	−0.77 (−0.86 to −0.69)	6297 (6041–6483)	13,113 (12,383–13,661)	7.04 (6.68–7.27)	5.07 (4.78–5.29)	−0.95 (−1 to −0.89)	177,724 (172,210–182,607)	348,544 (331,533–362,398)	181.09 (174.39–186.12)	132.10 (125.59–137.34)	−1.03 (−1.13 to −0.93)
Western Europe	43,785 (42,523–44,808)	76,360 (71,329–79,497)	8.13 (7.9–8.32)	9.24 (8.72–9.58)	0.56 (0.37–0.74)	27,288 (26,180–28,012)	34,397 (31,525–36,115)	4.80 (4.62–4.92)	3.65 (3.41–3.81)	−0.72 (−0.8 to −0.64)	651,147 (632,893–665,509)	712,093 (672,401–741,301)	121.21 (118–123.91)	85.44 (81.46–88.58)	−1.2 (−1.29 to −1.11)
Western sub-Saharan Africa	3273 (2623–4025)	11,872 (8842–14,297)	3.52 (2.84–4.3)	5.59 (4.18–6.7)	2.04 (1.84–2.25)	2421 (1964–2948)	8494 (6355–10,192)	2.86 (2.34–3.44)	4.58 (3.43–5.43)	1.45 (1.22–1.67)	66,215 (53,020–81,477)	235,235 (175,348–283,946)	71.08 (57.33–86.89)	110.00 (82.38–132.2)	1.95 (1.75–2.15)

ASR = age-standardized rate, CI = confidence interval, DALY = disability-adjusted life year, EAPC = estimated annual percentage change, GBD = Global Burden of Disease, SDI = socio-demographic index, UI = uncertainty interval.

In 2021, among the 21 GBD regions, the region with the most severe ASR of EC was East Asia. But from 1990 to 2021, the region with the most significant decline in the ASR of EC was Central Asia, and the EAPC of the ASR was all <−3. Notably, against the general trend of the decline in EC burden in most regions of the world, the ASPR in high SDI regions showed a trend of increasing year by year (EAPC = 0.48 [95% CI: 0.19–0.78]), such as Western Europe EAPC = 0.56 (95% CI: 0.37–0.74) and high-income Asia Pacific (EAPC = 0.44 [95% CI: 0.07–0.81]). In addition, Figure 1G–I shows that the 3 ASRs of EC in some countries in North America, Western Europe and North Africa still presented an upward trend.

From 1990 to 2021, in the EC analysis of ASPR, ASDR, and ASDALYR with SDI, among the 21 GBD regions, the low–middle SDI and middle SDI regions deviated more from the flat fitted values. The specific GBD regions were East Asia and Southern Sub-Saharan Africa (Fig. 1J–L). Moreover, in the national-level analysis, it was readily observable that the ASR of EC reached its lowest point in countries with medium-high SDI (Supplementary Material 1A–C, Supplemental Digital Content 1).

Moreover, the prediction outcomes of the BAPC model demonstrated that the number of prevalent EC cases escalated from 0.87 million cases in 2021 to 0.91 million cases in 2050; the number of deaths declined from 0.47 million cases to 0.45 million cases; the number of DALYs cases decreased from 11.25 million cases to 10.31 million cases. Additionally, the ASPR declined from 11.39 per 100,000 in 2021 to 9.85 per 100,000, a 13.50% reduction; the ASDR decreased from 6.19 per 100,000 to 4.85 per 100,000, a 21.65% decrease; the ASDALYR dropped from 147.29 per 100,000 to 111.32 per 100,000, a 24.42% decline (Fig. 1M–O). All results predicted by BAPC and their confidence intervals are provided in Supplementary Table, Supplemental Digital Content 4. While the current prevalence of cases continues to rise, the overall disease burden of EC shows a downward trend. However, the baseline burden of EC remains elevated.

The 3rd-level risk factors for esophageal cancer in GBD 2021 include smoking, low-vegetable diets, and chewing tobacco, with smoking being the most significant contributor. Globally, eliminating smoking as a risk factor could reduce esophageal cancer deaths by 37.99%. The most notable GBD regions demonstrating this effect are East Asia (age-standardized PAF [ASPAF]: 46.25%), high-income North America (ASPAF: 45.16%), and high-income Asia-Pacific (ASPAF: 41.21%), all exceeding the global average. In South Asia, chewing tobacco remains the most impactful risk factor for EC, with an ASPAF of 22.85%. The trend in risk factor analysis for EC DALYs follows the same pattern (Supplementary Material 2A–C, Supplemental Digital Content 2).

From 1990 to 2021, East Asia exhibited the largest overall increase in esophageal cancer prevalent cases (305,905,570.9), with population growth contributing 484,088,01.82 (15.82%), population aging contributing 155,602,507.7 (50.87%), and epidemiological changes contributing 101,893,461.4 (33.31%). South Asia had the 2nd largest overall increase (79,169,023.82), driven by population growth (274,433,453.56, 34.66%), population aging (171,623,292.55, 21.68%), and epidemiological changes (34,563,177.71, 43.66%). Additionally, the trends in deaths and DALYs were similar to those of prevalence. (Supplementary Material 3A–C, Supplemental Digital Content 3)

### 3.2. Stomach cancer

**Figure 2.**
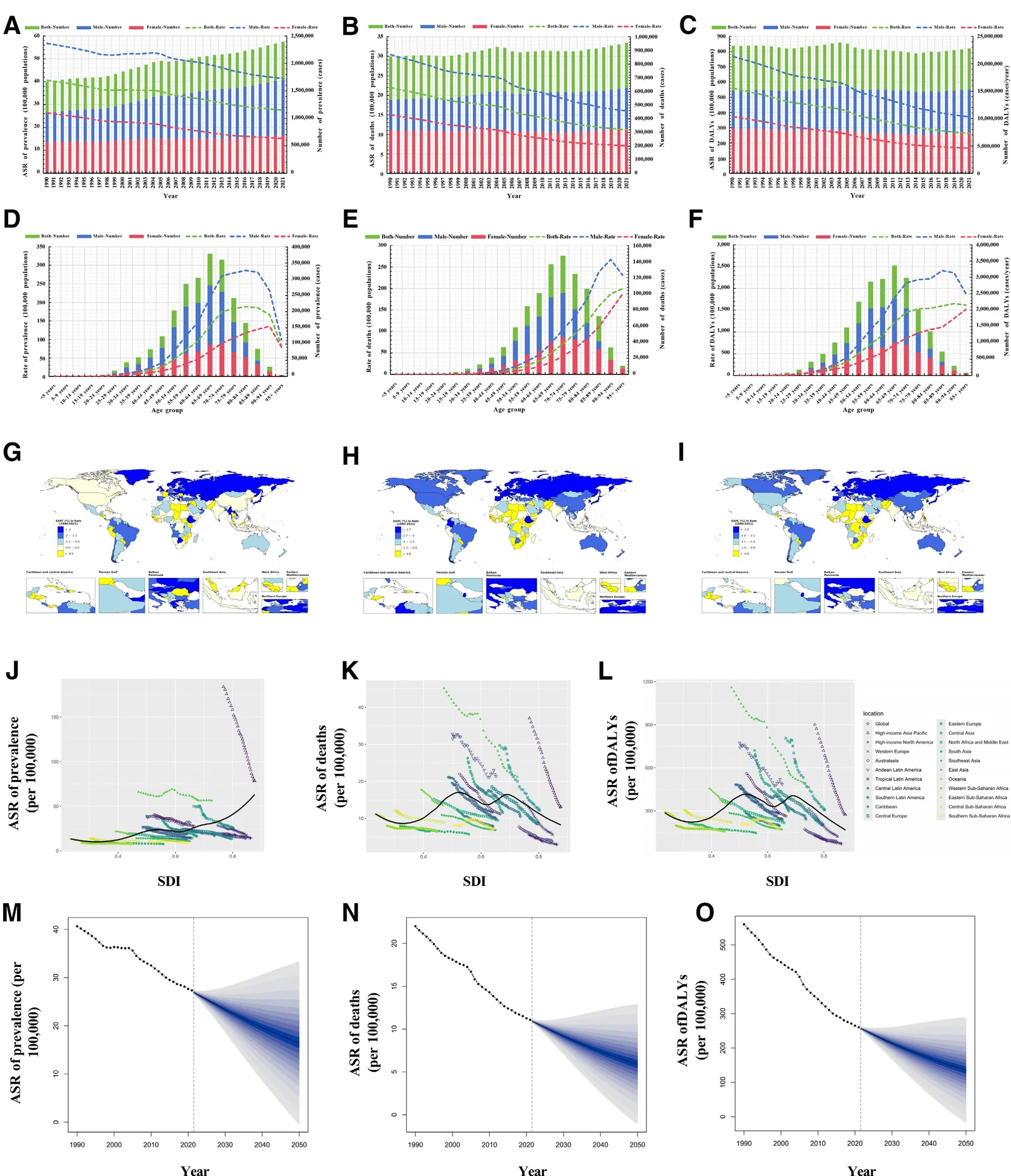
Trends in disease burden of SC (1990–2050). (A–C) Show prevalence, deaths, and DALYs burden trends for SC across different years and sexes from 1990 to 2021; (D–F) present age-specific and sex-disaggregated trends for SC’s prevalence, deaths, and DALYs burden during the same period; (G–I) display global mapping of SC’s prevalence, deaths, and DALYs using the EAPC; (J–L) illustrate ASR of prevalence, deaths, and DALYs across 21 GBD regions with varying SDI; and (M–O) present ASR of prevalence, deaths, and DALYs with projected 2050 projections from the BAPC model. ASR = age-standardized rate, BAPC = Bayesian age-period-cohort, DALYs = disability-adjusted life years, EAPC = estimated annual percentage change, GBD = Global Burden of Disease, SC = stomach cancer, SDI = socio-demographic index.

Globally, the number of prevalent cases of SC increased from 0.17 million cases in 1990 to 0.24 million cases in 2021, representing a 41.18% rise; the number of deaths increased from 0.09 million cases to 0.10 million cases, an 11.11% increment. However, the number of DALYs decreased from 2.32 million cases to 2.28 million cases, a 1.72% decline (Table 2). The global burden of SC among males exceeded that among females across all metrics, with the most prominent disparity observed in the ASPR, where the male rate was 2.72 times that of females (Fig. 2A–C). Consistent with the trend observed for EC, the ASPR, ASDR, and ASDALYR of SC all exhibited a downward trend from 1990 to 2021. The ASDALYR showed the most significant decrease, declining from 559.72 per 100,000 (95% UI: 499.09–615.77) in 1990 to 262.75 per 100,000 (95% UI: 226.08–301.02) in 2021 (EAPC = −2.54 [95% CI: −2.62 to −2.46]). SC also demonstrated a trend of increasing burden with advancing age in the age group older than 50 years (Fig. 2D–F).

**Table 2:** Trends in stomach cancer burden across 5 SDI and 22 GBD regions.

Location	Prevalence					Deaths					DALYs				
	Number, 1990 (95% UI)	Number, 2021, (95% UI)	ASR, 1990 (95% UI)	ASR, 2021 (95% UI)	EAPC, 1990–2021 (95% CI)	Number, 1990 (95% UI)	Number, 2021 (95% UI)	ASR, 1990 (95% UI)	ASR, 2021 (95% UI)	EAPC, 1990–2021 (95% CI)	Number, 1990 (95% UI)	Number, 2021 (95% UI)	ASR, 1990 (95% UI)	ASR, 2021 (95% UI)	EAPC, 1990–2021 (95% CI)
High–middle SDI	500,512 (445,395–544,263)	749,747 (610,195–903,200)	48.66 (43.46–52.91)	38.28 (31.13–46.09)	−0.72 (−0.84 to −0.61)	304,773 (274,911–330,960)	295,105 (244,787–343,611)	31.08 (28.09–33.7)	14.93 (12.4–17.36)	−2.47 (−2.59 to −2.35)	8,241,238 (7,281,203–9,010,822)	6,901,607 (5,703,610–8,141,888)	802.75 (711.79–876.76)	353.18 (291.89–416.78)	−2.79 (−2.91 to −2.66)
High SDI	592,944 (569,778–608,932)	592,767 (542,982–629,774)	54.75 (52.72–56.23)	29.39 (27.2–31.24)	−1.99 (−2.1 to −1.89)	175,683 (166,255–181,197)	153,539 (136,670–165,354)	15.86 (15.01–16.37)	6.83 (6.18–7.34)	−2.79 (−2.81 to −2.76)	4,099,422 (3,902,053–4,218,897)	2,897,404 (2,654,584–3,101,177)	381.13 (362.21–392.23)	146.10 (135.56–155.89)	−3.15 (−3.18 to −3.13)
Low–middle SDI	88,349 (76,931–106,573)	157,615 (139,220–179,612)	13.06 (11.48–15.88)	10.34 (9.11–11.79)	−0.69 (−0.73 to −0.66)	62,598 (54,993–76,278)	107,456 (94,064–122,211)	10.45 (9.25–12.79)	7.71 (6.69–8.76)	−0.89 (−0.94 to −0.85)	1,874,805 (1,629,366–2,261,562)	2,953,125 (2,599,859–3,357,038)	274.45 (239.89–333.49)	192.56 (169.42–219.14)	−1.08 (−1.12 to −1.04)
Low SDI	35,766 (28,501–40,942)	57,598 (45,194–65,610)	14.15 (11.27–16.18)	10.22 (8.07–11.56)	−1.1 (−1.15 to −1.05)	26,410 (21,016–30,213)	41,192 (32,515–46,864)	11.90 (9.41–13.57)	8.46 (6.71–9.6)	−1.1 (−1.15 to −1.04)	795,640 (636,078–910,967)	1,198,903 (940,407–1,371,290)	311.98 (248.62–357.31)	209.77 (165.6–238.95)	−1.35 (−1.4 to −1.29)
Middle SDI	452,701 (385,138–524,307)	834,461 (673,685–1,015,061)	39.87 (34.3–45.96)	30.06 (24.25–36.54)	−0.93 (−1.01 to −0.84)	284,016 (246,760–329,474)	356,459 (294,160–423,770)	28.10 (24.61–32.65)	13.72 (11.31–16.22)	−2.42 (−2.54 to −2.3)	8,208,596 (7,034,443–9,506,074)	8,820,717 (7,336,337–10,567,624)	721.81 (624.06–835.38)	320.24 (266.07–382.87)	−2.75 (−2.86 to −2.63)
Andean Latin America	8367 (7331–9493)	18,454 (14,899–22,663)	38.96 (34.2–44.33)	30.94 (24.9–37.99)	−0.92 (−1.06 to −0.78)	6455 (5696–7324)	12,368 (10,039–15,114)	32.80 (28.93–37.32)	21.33 (17.3–26.08)	−1.34 (−1.45 to −1.22)	168,541 (147,325–190,607)	292,675 (235,472–359,119)	773.16 (678.85–875.03)	485.41 (391.23–595.07)	−1.73 (−1.88 to −1.59)
Australasia	4777 (4484–5078)	7317 (6521–7977)	20.30 (19.04–21.54)	14.35 (12.88–15.6)	−1.16 (−1.25 to −1.08)	1804 (1690–1906)	2191 (1921–2388)	7.73 (7.25–8.16)	3.91 (3.49–4.25)	−2.49 (−2.61 to −2.38)	39,867 (37,637–41,910)	42,612 (38,796–46,055)	171.48 (161.92–180.59)	84.88 (77.89–91.35)	−2.26 (−2.38 to −2.15)
Caribbean	4259 (3913–4671)	6225 (5418–7091)	16.00 (14.72–17.52)	11.65 (10.14–13.28)	−0.92 (−0.99 to −0.86)	3138 (2873–3450)	4191 (3626–4808)	12.38 (11.34–13.57)	7.79 (6.74–8.94)	−1.68 (−1.76 to −1.59)	79,115 (71,374–87,961)	104,402 (89,158–120,825)	296.37 (268.46–329.4)	195.67 (167.08–226.69)	−1.25 (−1.35 to −1.16)
Central Asia	17,869 (17,097–18,804)	14,216 (12,781–16,004)	35.90 (34.25–37.85)	16.18 (14.62–18.13)	−2.33 (−2.44 to −2.21)	12,366 (11,762–13,059)	9332 (8400–10,425)	26.28 (24.93–27.77)	11.56 (10.46–12.83)	−2.36 (−2.43 to −2.29)	359,741 (343,480–378,737)	264,445 (236,310–298,863)	720.77 (687.22–760.05)	298.62 (268.02–336.43)	−2.66 (−2.74 to −2.59)
Central Europe	36,118 (34,733–37,414)	29,450 (27,058–32,038)	23.98 (23.06–24.82)	13.89 (12.78–15.08)	−1.76 (−1.88 to −1.63)	27,224 (26,133–28,216)	19,182 (17,694–20,668)	18.50 (17.72–19.19)	8.54 (7.89–9.22)	−2.47 (−2.53 to −2.41)	666,468 (641,593–689,338)	418,848 (387,111–452,190)	444.41 (427.88–459.51)	201.30 (185.93–217.26)	−2.64 (−2.73 to −2.55)
Central Latin America	23,110 (22,398–23,756)	47,626 (42,488–53,819)	26.39 (25.46–27.16)	18.72 (16.71–21.15)	−1.36 (−1.44 to −1.28)	16,459 (15,832–16,952)	28,763 (25,594–32,289)	21.14 (20.17–21.8)	11.69 (10.41–13.09)	−2.1 (−2.15 to −2.05)	427,467 (414,311–439,042)	722,803 (644,910–813,721)	482.23 (466.65–496.63)	282.56 (252.21–317.88)	−1.99 (−2.07 to −1.9)
Central sub-Saharan Africa	3470 (2563–4236)	6562 (4888–8198)	13.84 (10.27–16.76)	10.54 (7.83–12.99)	−0.91 (−0.96 to −0.87)	2570 (1888–3136)	4607 (3474–5734)	12.13 (9.06–14.67)	8.93 (6.76–11.06)	−1.01 (−1.04 to −0.99)	77,401 (57,088–94,838)	139,267 (104,199–175,157)	305.95 (225.6–372.66)	220.04 (165.52–273.38)	−1.11 (−1.14 to −1.07)
East Asia	628,113 (515,535–732,748)	1,248,669 (966,817–1,569,812)	66.04 (54.57–76.88)	56.31 (43.73–70.59)	−0.48 (−0.64 to −0.32)	381,435 (318,259–449,024)	455,947 (355,895–567,156)	45.18 (38.27–53.36)	21.26 (16.59–26.22)	−2.28 (−2.42 to −2.15)	10,985,613 (9,060,114–12,871,606)	10,921,589 (8,491,323–13,670,612)	1160.15 (965.28–1363)	496.94 (386.19–619.66)	−2.87 (−3.05 to −2.68)
Eastern Europe	141,284 (137,836–144,022)	85,376 (78,555–92,209)	50.04 (48.83–51.02)	25.17 (23.11–27.17)	−2.35 (−2.46 to −2.23)	83,348 (81,061–85,100)	42,700 (39,316–46,074)	29.71 (28.85–30.32)	12.15 (11.17–13.12)	−2.8 (−2.91 to −2.68)	2,264,040 (2,215,067–2,307,995)	1,031,465 (943,209–1,119,000)	805.45 (787.5–821.16)	306.55 (280.01–332.59)	−3.4 (−3.52 to −3.29)
Eastern sub-Saharan Africa	11,150 (8829–12,708)	16,378 (13,360–19,226)	13.09 (10.38–14.98)	8.48 (6.99–9.93)	−1.62 (−1.7 to −1.55)	8233 (6531–9437)	11,542 (9518–13,490)	11.09 (8.81–12.7)	7.09 (5.92–8.28)	−1.33 (−1.43 to −1.23)	252,008 (199,160–287,489)	344,564 (281,270–404,252)	292.75 (232.08–335.22)	175.48 (144.38–205.24)	−1.9 (−1.99 to −1.82)
Global	1,671,262 (1,524,321–1,796,161)	2,393,213 (2,059,698–2,771,476)	40.64 (37.25–43.68)	27.58 (23.75–31.89)	−1.24 (−1.32 to −1.16)	854,185 (772,885–939,973)	954,374 (821,751–1,089,577)	22.01 (20.03–24.19)	11.20 (9.62–12.73)	−2.15 (−2.2 to −2.09)	23,237,292 (20,605,349–25,526,194)	22,786,633 (19,576,344–26,118,869)	559.72 (499.09–615.77)	262.75 (226.08–301.02)	−2.54 (−2.62 to −2.46)
High-income Asia Pacific	378,489 (362,050–391,739)	339,616 (302,557–365,926)	184.07 (175.58–190.55)	78.49 (71.73–84.73)	−2.74 (−2.83 to −2.65)	72,724 (67,979–75,561)	70,689 (60,268–77,102)	37.06 (34.46–38.6)	13.13 (11.52–14.14)	−3.45 (−3.48 to −3.42)	1,831,592 (1,693,439–1,906,727)	1,194,021 (1,061,152–1,287,243)	900.48 (832.19–937.87)	273.07 (250.22–294.45)	−3.91 (−3.95 to −3.86)
High-income North America	63,842 (60,830–65,737)	87,177 (81,936–90,901)	18.45 (17.65–18.97)	14.34 (13.54–14.9)	−0.81 (−0.92 to −0.7)	19,836 (18,496–20,571)	19,363 (17,658–20,393)	5.57 (5.21–5.77)	2.95 (2.72–3.1)	−2.11 (−2.14 to −2.07)	438,698 (419,948–450,664)	419,798 (396,159–437,428)	129.31 (124.2–132.72)	70.88 (67.42–73.54)	−1.97 (−2.04 to −1.9)
North Africa and Middle East	35,512 (26,533–40,046)	68,772 (47,005–78,458)	19.20 (14.33–21.66)	14.18 (9.74–16.14)	−0.91 (−1.01 to −0.8)	24,484 (18,566–27,726)	39,717 (27,883–45,110)	15.19 (11.51–17.15)	9.43 (6.7–10.67)	−1.48 (−1.54 to −1.43)	707,934 (531,826–799,708)	1,057,332 (732,444–1,215,400)	380.20 (288.74–429.88)	217.41 (151.81–247.77)	−1.75 (−1.82 to −1.67)
Oceania	755 (524–981)	1536 (1188–1920)	21.73 (15.73–27.91)	17.54 (13.73–21.81)	−0.73 (−0.8 to −0.65)	497 (358–641)	957 (744–1201)	17.76 (13.08–22.53)	13.45 (10.67–16.61)	−0.94 (−0.98 to −0.9)	15,969 (11,130–20,718)	30,296 (23,279–38,275)	456.41 (329.1–588.19)	341.81 (265.8–428.05)	−0.93 (−1.02 to −0.85)
South Asia	66,073 (56,413–82,636)	118,153 (103,186–143,236)	10.02 (8.57–12.72)	7.53 (6.55–9.1)	−0.87 (−0.95 to −0.79)	46,010 (39,093–58,610)	82,924 (71,235–99,367)	7.88 (6.68–10.17)	5.72 (4.91–6.84)	−0.96 (−1.01 to −0.91)	1,432,660 (1,232,646–1,789,557)	2,295,875 (2,004,284–2,773,948)	215.57 (183.72–273.18)	145.55 (126.31–175.43)	−1.21 (−1.28 to −1.14)
Southeast Asia	40,283 (32,292–46,230)	75,213 (63,996–87,890)	14.10 (11.34–16.16)	10.79 (9.23–12.59)	−1.06 (−1.14 to −0.98)	27,448 (22,187–31,331)	43,303 (37,510–51,160)	10.92 (8.91–12.54)	6.83 (5.94–8.16)	−1.53 (−1.59 to −1.47)	820,413 (659,024–941,613)	1,193,413 (1,021,566–1,404,696)	285.40 (229.89–326.55)	170.78 (146.62–201.09)	−1.83 (−1.9 to −1.76)
Sout−ern Latin America	11,436 (10,824–12,090)	14,493 (13,239–15,762)	24.51 (23.17–25.92)	16.94 (15.48–18.42)	−1.01 (−1.13 to −0.88)	8435 (7955–8911)	9292 (8379–10,092)	18.65 (17.56–19.75)	10.51 (9.47–11.41)	−1.57 (−1.64 to −1.5)	201,215 (190,126–212,735)	205,763 (188,547–223,240)	432.39 (408.29–457.03)	241.14 (221.13–261.57)	−1.71 (−1.82 to −1.6)
Southern sub-Saharan Africa	3079 (2447–3446)	5557 (4643–6309)	10.27 (8.09–11.5)	8.90 (7.39–10.03)	−0.47 (−0.74 to −0.2)	2151 (1697–2411)	3967 (3307–4472)	8.13 (6.38–9.11)	7.13 (5.92–7.98)	−0.51 (−0.73 to −0.3)	62,836 (49,869–70,324)	111,785 (94,036–128,129)	208.13 (164.69–233.47)	177.66 (148.51–201.91)	−0.5 (−0.84 to −0.15)
Tropical Latin America	23,116 (22,233–23,920)	37,355 (35,278–39,176)	24.07 (22.97–24.99)	14.39 (13.57–15.09)	−1.79 (−1.84 to −1.73)	16,803 (16,029–17,446)	24,867 (23,070–26,204)	19.54 (18.45–20.37)	9.78 (9.05–10.32)	−2.25 (−2.29 to −2.21)	449,287 (432,709–464,461)	619,369 (586,946–648,930)	463.86 (444.88–480.64)	237.81 (225.12–249.26)	−2.28 (−2.33 to −2.22)
Western Europe	161,528 (153,263–167,398)	148,755 (135,516–158,088)	28.45 (27.08–29.41)	17.25 (15.97–18.24)	−1.41 (−1.5 to −1.32)	86,059 (80,686–89,117)	56,322 (49,569–60,192)	14.52 (13.64–15.03)	5.57 (5.02–5.9)	−3.16 (−3.21 to −3.1)	1,769,700 (1,688,901–1,823,382)	1,038,057 (950,524–1,095,124)	316.11 (302.86–324.92)	120.38 (112.31–126.18)	−3.08 (−3.16 to −3)
Western sub-Saharan Africa	8632 (7279–10,108)	16,314 (12,399–19,471)	9.16 (7.78–10.77)	7.64 (5.93–8.99)	−0.39 (−0.47 to −0.31)	6705 (5699–7866)	12,151 (9468–14,304)	8.01 (6.85–9.5)	6.69 (5.34–7.9)	−0.55 (−0.62 to −0.48)	186,729 (157,190–218,063)	338,256 (257,059–405,471)	196.52 (166.83–230.65)	156.08 (120.91–184.46)	−0.55 (−0.62 to −0.48)

ASR = age-standardized rate, CI = confidence interval, DALY = disability-adjusted life year, EAPC = estimated annual percentage change, GBD = Global Burden of Disease, SDI = socio-demographic index, UI = uncertainty interval.

In 2021, among the 5 SDI regions, the most severe ASR of SC was concentrated in the high–middle SDI region. In 2021, among the 21 GBD regions, the region with the highest ASPR was high-income Asia Pacific (78.49 per 100,000 [95% UI: 71.73–84.73]), the region with the highest ASDR was Andean Latin America (21.33 per 100,000 [95% UI: 17.3–26.08]), and the region with the highest ASDALYR was East Asia (496.94 per 100,000 [95% UI: 386.19–619.66]) (Table 2). Among the 204 countries, only some countries in North America and Africa showed positive EAPC values for SC, while the ASR of SC exhibited an overall downward trend globally (Fig. 2G–I). Notably, in the analysis of SC and SDI, high-income Asia Pacific, as a high SDI region, had a relatively high ASPR. At the same SDI level, only high-income Asia Pacific was above the fitted value of ASPR (Fig. 2J–L). The same SDI trend can also be observed across 204 countries (Supplementary Material 1D–F, Supplemental Digital Content 1).

Furthermore, the BAPC model indicated that the number of prevalent cases in SC would decrease from 2.09 million cases in 2021 to 1.52 million cases in 2050, a decrease of 27.27%. The number of deaths would decline from 0.85 million cases to 0.55 million cases, a 35.29% decrement; DALYs would decrease from 19.87 million cases to 12.55 million cases, a 36.84% decline. Moreover, the ASPR would decline from 27.33 per 100,000 in 2021 to 16.43 per 100,000 in 2050, a 39.90% reduction; the ASDR would decrease from 11.07 per 100,000 to 5.91 per 100,000, a 46.60% decrease; and the ASDALYR would drop from 260.23 per 100,000 to 135.47 per 100,000, a 47.94% decline (Fig. 2M–O). All results predicted by BAPC and their CIs are provided in Supplementary Table, Supplemental Digital Content 4. Consequently, despite the predicted nearly 2-fold decrease in the ASR of SC, the existing case base of SC remains substantial.

The 3rd-tier risk factors for SC in GBD 2021 include a diet high in sodium and smoking. Globally, eliminating the diet high in sodium risk factor could reduce SC-related deaths by 7.93%. Central Europe shows the strongest impact of a diet high in sodium on SC deaths (ASPAF: 8.31%). Similarly, eliminating smoking exposure worldwide could decrease SC deaths by 11.18%, with East Asia being the most affected region (ASPAF: 14.28%). The trend in SC-related DALYs risk factors mirrors that of deaths (Supplementary Material 2C–D, Supplemental Digital Content 2).

From 1990 to 2021, East Asia exhibited the largest overall increase in both SC prevalent cases (719,777,810.4) and deaths (155,518,065.8). For prevalence, population aging contributed 314,364,894.2 (43.68%), epidemiological changes contributed 296,942,442.9 (41.25%), and population growth contributed 108,470,473.3 (15.07%); for deaths, population aging contributed 128,269,307.6 (82.48%), population growth contributed 39,030,947.82 (25.1%), and epidemiological changes contributed −11,782,189.6 (−7.58%). Southeast Asia had the 2nd largest increase in prevalence (167,387,862.57), driven by epidemiological changes (63,547,436.62, 37.96%), population aging (53,030,494.9, 31.68%), and population growth (50,809,931.05, 30.35%), while South Asia had the 2nd largest increase in deaths (65,269,527.47), driven by population growth (265,738,834.2, 40.71%), epidemiological changes (21,012,311.49, 32.19%), and population aging (17,683,381.78, 27.09%). The trend in DALY decomposition is similar to that of deaths (Supplementary Material 3D–F, Supplemental Digital Content 3).

### 3.3. Colon and rectal cancer

**Figure 3.**
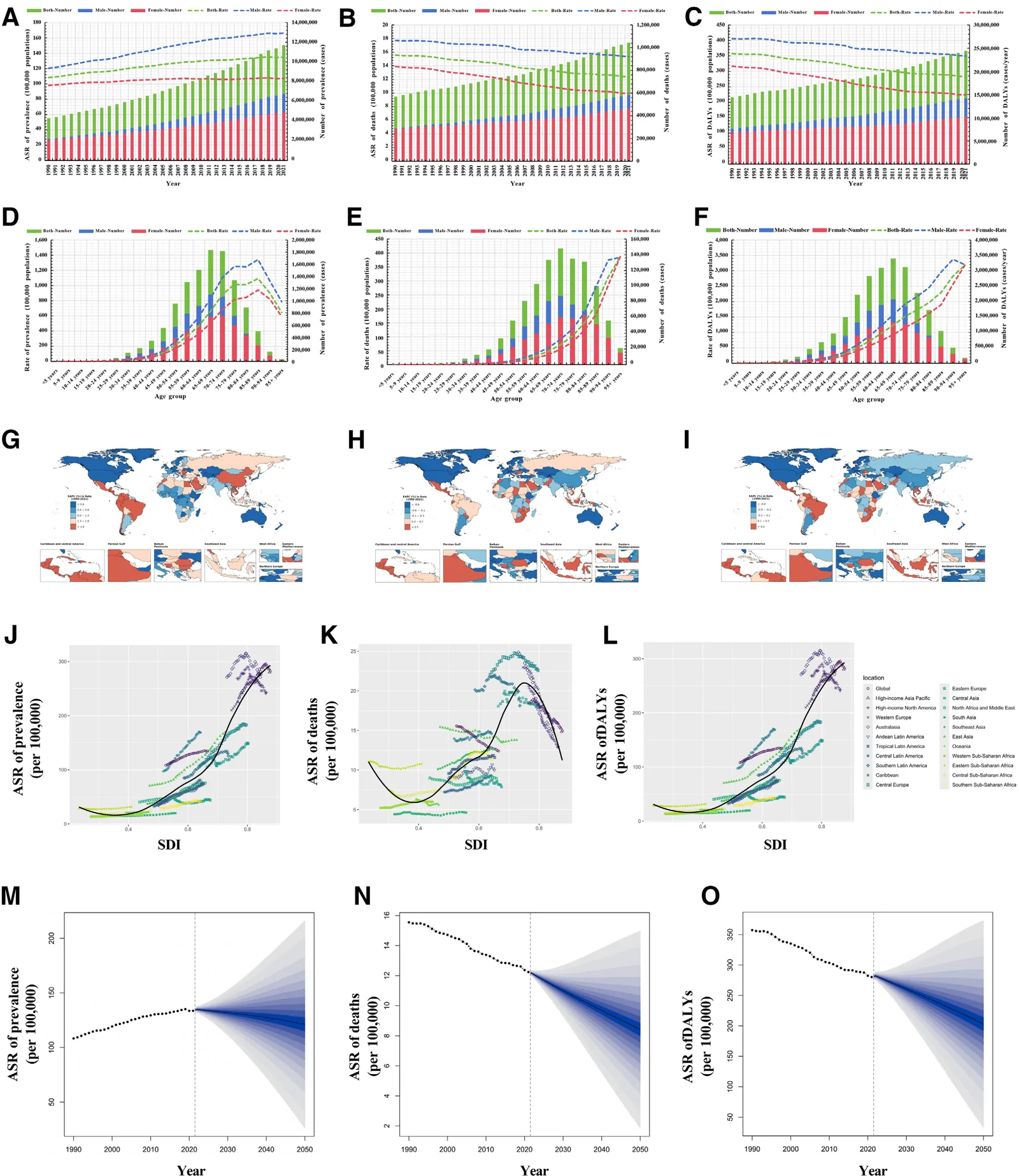
Trends in disease burden of CRC (1990–2050). (A–C) Show prevalence, deaths, and DALYs burden trends for CRC across different years and sexes from 1990 to 2021; (D–F) present age-specific and sex-disaggregated trends for CRC’s prevalence, deaths, and DALYs burden during the same period; (G–I) display global mapping of CRC’s prevalence, deaths, and DALYs using the EAPC; (J–L) illustrate ASR of prevalence, deaths, and DALYs across 21 GBD regions with varying SDI; and (M–O) present age-standardized rates of prevalence, deaths, and DALYs with projected 2050 projections from the BAPC model. ASR = age-standardized rate, BAPC = Bayesian age-period-cohort, CRC = colon and rectal cancer, DALYs = disability-adjusted life years, EAPC = estimated annual percentage change, GBD = Global Burden of Disease, SDI = socio-demographic index.

Globally, the number of prevalent cases of CRC increased from 0.43 million cases in 1990 to 1.17 million cases in 2021, representing a 172.09% rise; the number of deaths rose from 0.06 million cases to 0.10 million cases, a 66.67% increase; and the number of DALYs increased from 1.4 million cases to 2.4 million cases, a 71.43% growth (Table 3). In contrast to EC and SC, the ASPR of CRC increased from 108.25 per 100,000 (95% UI: 103.29–112.75) in 1990 to 134.84 per 100,000 (95% UI: 124.21–144.77) in 2021 (EAPC = 0.74 [95% CI: 0.69–0.78]). Over time, the burden of CRC in males consistently exceeded that in females (Fig. 3A–C). Consistent with EC and SC, the burden of CRC was more pronounced in the age group older than 50 years (Fig. 3E–G).

**Table 3:** Trends in colon and rectal cancer burden across 5 SDI and 22 GBD rregions.

Location	Prevalence					Deaths					DALYs				
	Number, 1990 (95% UI)	Number, 2021, (95% UI)	ASR, 1990 (95% UI)	ASR, 2021 (95% UI)	EAPC, 1990–2021 (95% CI)	Number, 1990 (95% UI)	Number, 2021, (95% UI)	ASR, 1990 (95% UI)	ASR, 2021 (95% UI)	EAPC, 1990–2021 (95% CI)	Number, 1990 (95% UI)	Number, 2021, (95% UI)	ASR, 1990 (95% UI)	ASR, 2021 (95% UI)	EAPC, 1990–2021 (95% CI)
High–middle SDI	1,079,219 (1,023,078–1,134,286)	3,586,357 (3,202,964–4,010,443)	106.77 (101.13–112.27)	182.09 (162.66–203.94)	1.85 (1.8–1.91)	171,222 (160,927–179,670)	308,175 (278,042–338,271)	18.12 (17.02–19.01)	15.71 (14.14–17.25)	−0.52 (−0.59 to −0.46)	4,416,013 (4,153,232–4,663,023)	7,079,678 (6,397,925–7,847,048)	436.82 (410.01–460.37)	364.61 (329.26–404.77)	−0.69 (−0.74 to −0.64)
High SDI	2,486,772 (2,382,582–2,573,169)	4,895,851 (4,571,265–5,117,055)	225.32 (216.26–232.99)	245.13 (231.33–255.18)	0.26 (0.14–0.38)	243,217 (227,488–251,652)	336,566 (299,022–359,613)	21.86 (20.43–22.64)	15.02 (13.58–15.92)	−1.31 (−1.34 to −1.27)	5,344,611 (5,120,390–5,504,892)	6,705,749 (6,179,256–7,070,831)	490.50 (470.41–505.01)	338.23 (316.75–354.91)	−1.28 (−1.32 to −1.25)
Low–middle SDI	128,613 (112,149–145,141)	460,178 (419,953–506,242)	19.12 (16.84–21.45)	29.93 (27.42–32.87)	1.5 (1.45–1.56)	33,887 (29,282–38,356)	91,274 (83,733–99,854)	5.72 (4.99–6.45)	6.55 (6.02–7.17)	0.48 (0.45–0.51)	1,021,299 (879,104–1,160,914)	2,564,121 (2,333,601–2,815,224)	149.42 (129.02–169.5)	166.47 (151.97–182.5)	0.37 (0.34–0.4)
Low SDI	48,619 (38,784–55,507)	121,918 (108,718–136,028)	19.69 (15.89–22.39)	21.93 (19.72–24.33)	0.25 (0.12–0.39)	15,462 (12,106–17,687)	31,848 (28,504–35,514)	7.21 (5.75–8.21)	6.88 (6.18–7.62)	−0.22 (−0.33 to −0.12)	452,673 (350,578–521,203)	901,645 (799,642–1,014,005)	181.87 (142.04–208.32)	162.29 (145.25–181.89)	−0.49 (−0.59 to −0.39)
Middle SDI	518,844 (470,526–572,470)	2,602,952 (2,277,687–2,947,760)	45.92 (41.83–50.58)	93.46 (81.91–105.55)	2.48 (2.38–2.59)	105,725 (95,513–116,511)	274,871 (243,369–306,694)	10.79 (9.76–11.82)	10.64 (9.42–11.84)	−0.1 (−0.13 to −0.06)	3,142,818 (2,820,037–3,490,094)	7,120,502 (6,308,564–7,920,507)	273.63 (246.6–302.13)	259.35 (230.17–288.24)	−0.22 (−0.27 to −0.17)
Andean Latin America	7460 (6505–8504)	44,440 (35,310–55,834)	35.34 (30.78–40.19)	74.64 (59.29–93.61)	2.63 (2.5–2.75)	1723 (1495–1967)	5778 (4597–7047)	8.88 (7.71–10.08)	10.00 (7.98–12.18)	0.42 (0.35–0.49)	43,988 (37,838–50,561)	136,183 (108,535–167,982)	203.47 (175.15–233.97)	226.76 (180.59–279.8)	0.39 (0.28–0.49)
Australasia	63,451 (58,696–68,073)	142,409 (127,043–159,798)	270.87 (250.84–290.21)	276.54 (248.21–309.1)	0.02 (−0.17–0.2)	5778 (5305–6246)	8276 (7179–9408)	24.88 (22.77–26.91)	14.62 (12.77–16.52)	−1.67 (−1.76 to −1.57)	133,623 (123,838–143,847)	166,740 (147,524–186,891)	577.93 (535.91–621.91)	327.19 (290.9–364.68)	−2.06 (−2.17 to −1.96)
Caribbean	25,784 (24,248–27,232)	90,731 (78,943–103,021)	97.50 (91.75–103.05)	168.78 (146.85–191.54)	1.98 (1.86–2.1)	3472 (3239–3721)	7882 (6867–8970)	13.96 (13.01–14.95)	14.57 (12.72–16.6)	0.28 (0.24–0.31)	83,680 (78,049–89,689)	179,752 (155,874–205,420)	317.26 (295.9–339.95)	335.02 (290.41–383.11)	0.33 (0.28–0.37)
Central Asia	23,820 (22,398–25,266)	38,034 (34,040–41,805)	48.01 (45.11–51.01)	43.76 (39.32–47.84)	0.08 (−0.17 to 0.33)	4831 (4499–5157)	6146 (5493–6772)	10.35 (9.61–11.06)	7.87 (7.06–8.65)	−0.6 (−0.71 to −0.5)	140,374 (132,361–149,146)	169,952 (151,537–187,708)	280.69 (263.92–298.74)	196.55 (175.59–216.68)	−0.88 (−0.99 to −0.76)
Central Europe	160,594 (153,408–168,047)	391,273 (361,471–423,445)	106.17 (101.49–111.09)	182.68 (168.91–197.59)	1.85 (1.66–2.05)	32,120 (30,603–33,396)	51,843 (47,752–55,681)	22.04 (21–22.93)	22.58 (20.81–24.28)	0.34 (0.21–0.46)	767,091 (735,174–795,936)	1,087,562 (1,003,626–1,168,378)	512.39 (489.92–531.48)	506.48 (467.97–544.57)	−0.12 (−0.24 to 0)
Central Latin America	28,622 (27,469–29,671)	207,018 (184,586–231,320)	32.62 (31.23–33.95)	81.17 (72.31–90.63)	2.99 (2.93–3.05)	5616 (5368–5819)	22,934 (20,342–25,521)	7.21 (6.85–7.49)	9.30 (8.26–10.35)	0.54 (0.44–0.64)	149,275 (143,941–154,015)	594,117 (529,274–662,367)	165.76 (159.24–171.33)	231.95 (206.64–258.57)	1.11 (1.03–1.18)
Central sub-Saharan Africa	4714 (3812–5783)	14,053 (10,834–18,389)	19.35 (15.86–23.41)	22.89 (17.85–29.79)	0.59 (0.38–0.81)	1494 (1200–1838)	3687 (2802–4932)	7.43 (6.05–9.04)	7.45 (5.77–10.14)	−0.15 (−0.25 to −0.05)	44,003 (35,103–54,650)	109,622 (82,688–146,971)	179.38 (145.64–220.18)	177.79 (135.17–238.59)	0.02 (−0.12 to 0.17)
East Asia	666,452 (578,648–761,268)	3,750,147 (3,055,407–4,488,997)	70.58 (61.63–80.23)	169.48 (138.42–202.5)	3.13 (3.02–3.24)	123,638 (106,930–141,857)	287,880 (235,559–343,280)	15.43 (13.39–17.61)	13.78 (11.33–16.35)	−0.45 (−0.48 to −0.41)	3,691,552 (3,152,648–4,246,182)	7,148,995 (5,822,935–8,561,079)	389.57 (334.25–446.9)	334.50 (274.01–399.93)	−0.59 (−0.66 to −0.51)
Eastern Europe	298,049 (286,577–309,651)	517,877 (479,461–558,304)	105.37 (101.36–109.62)	148.73 (137.67–160.33)	1.08 (0.91–1.25)	50,097 (48,178–51,688)	64,373 (59,115–69,822)	18.06 (17.33–18.65)	18.05 (16.58–19.56)	0.4 (0.2–0.59)	1,280,866 (1,237,775–1,320,907)	1,465,104 (1,343,981–1,600,585)	455.74 (440.17–469.53)	424.54 (389.69–463.67)	−0.48 (−0.61 to −0.35)
Eastern sub-Saharan Africa	23,226 (18,440–26,361)	57,913 (50,440–68,321)	28.51 (22.85–32.17)	31.57 (27.88–36.44)	0.19 (0.06–0.33)	7779 (5998–8844)	15,967 (13,940–18,320)	11.06 (8.71–12.49)	10.76 (9.41–12.15)	−0.17 (−0.23 to −0.12)	225,496 (171,119–258,759)	444,253 (385,800–525,458)	274.48 (211.05–312.21)	242.13 (211.3–279.08)	−0.58 (−0.68 to −0.48)
Global	4,266,770 (4,085,520–4,447,809)	11,679,120 (10,774,527–12,538,400)	108.25 (103.29–112.75)	134.84 (124.21–144.77)	0.74 (0.69–0.78)	570,319 (536,545–597,669)	1,044,072 (950,188–1,120,169)	15.56 (14.49–16.31)	12.40 (11.24–13.31)	−0.68 (−0.72 to −0.64)	14,396,658 (13,568,749–15,166,576)	24,401,100 (22,689,369–26,161,518)	357.33 (336.62–375.74)	283.24 (263.11–303.33)	−0.83 (−0.87 to −0.8)
High-income Asia Pacific	459,307 (440,006–477,176)	1,223,717 (1,095,145–1,300,805)	225.21 (215.11–234.21)	282.83 (258.21–298.68)	0.7 (0.59–0.8)	35,636 (33,643–36,954)	80,691 (67,267–88,001)	18.45 (17.23–19.2)	14.99 (13.03–16.09)	−0.41 (−0.5 to −0.31)	874,132 (837,041–905,135)	1,441,630 (1,272,496–1,549,759)	431.84 (411.77–447.55)	331.48 (303.85–352.47)	−0.93 (−0.98 to −0.88)
High-income North America	939,600 (894,734–980,496)	1,501,917 (1,412,159–1,563,567)	268.66 (256.73–279.93)	242.89 (229.88–252.06)	−0.41 (−0.54 to −0.28)	73,964 (67,846–77,460)	85,865 (77,871–90,928)	20.58 (18.95–21.52)	12.95 (11.89–13.65)	−1.54 (−1.59 to −1.49)	1,613,202 (1,529,313–1,679,101)	1,907,928 (1,788,111–2,003,824)	469.38 (446.3–487.53)	316.04 (298.52–330.84)	−1.36 (−1.41 to −1.31)
North Africa and Middle East	74,002 (63,328–83,045)	364,946 (321,981–411,865)	40.41 (35.24–44.98)	75.52 (66.69–85.42)	2.26 (2.13–2.39)	14,108 (12,126–15,839)	37,395 (32,759–42,274)	8.99 (7.94–10.06)	8.95 (7.85–10.08)	0.07 (−0.02 to 0.17)	409,788 (341,302–464,924)	1,012,652 (886,199–1,154,503)	220.98 (187.75–248.96)	209.04 (183.16–237.28)	−0.01 (−0.14 to 0.12)
Oceania	695 (576–833)	1807 (1540–2097)	21.00 (17.79–24.5)	21.41 (18.5–24.56)	0.03 (−0.06 to 0.12)	165 (136–200)	382 (324–446)	6.31 (5.37–7.42)	5.58 (4.8–6.42)	−0.4 (−0.47 to −0.34)	5153 (4142–6362)	11,643 (9764–13,750)	154.39 (127.38–186.63)	136.65 (116.1–159.45)	−0.39 (−0.48 to −0.3)
South Asia	91,741 (79,877–103,573)	312,461 (283,042–350,426)	14.05 (12.23–15.9)	19.69 (17.89–22.01)	0.96 (0.81–1.11)	25,281 (21,597–28,741)	66,943 (60,197–74,844)	4.42 (3.76–5.06)	4.63 (4.17–5.16)	0.06 (0–0.12)	787,251 (675,898–889,380)	1,908,668 (1,711,982–2,155,055)	119.12 (101.91–135.16)	120.37 (108.3–135.3)	−0.1 (−0.2–0.01)
Southeast Asia	104,516 (89,925–117,295)	499,194 (433,063–562,668)	37.09 (32.27–41.31)	71.39 (62.36–80.56)	2.15 (2.12–2.18)	24,777 (20,969–28,065)	79,420 (68,450–89,290)	10.11 (8.66–11.38)	12.74 (11.12–14.26)	0.79 (0.75–0.83)	735,822 (612,422–841,712)	2,166,650 (1,868,880–2,456,350)	257.93 (218.13–292.17)	313.37 (270.75–353.54)	0.61 (0.55–0.67)
Southern Latin America	39,512 (36,294–42,806)	107,741 (96,274–119,133)	84.86 (78–91.99)	125.45 (112.22–138.83)	1.54 (1.38–1.7)	8974 (8274–9706)	16,117 (14,307–18,002)	20.13 (18.48–21.81)	18.13 (16.12–20.25)	0.09 (−0.02 to 0.2)	206,237 (189,818–224,031)	349,581 (311,369–391,893)	445.86 (410.18–484.75)	407.99 (363.05–457.56)	−0.04 (−0.19 to 0.1)
Southern sub-Saharan Africa	8351 (7502–9935)	26,404 (23,841–29,510)	28.86 (25.84–34.87)	42.79 (38.69–47.32)	1.49 (1.39–1.59)	2225 (1974–2720)	6130 (5550–6786)	8.82 (7.79–10.94)	11.47 (10.37–12.61)	0.77 (0.59–0.96)	60,914 (54,662–72,328)	166,062 (149,959–186,753)	207.84 (185.42–251.15)	270.64 (244.94–301.85)	1.06 (0.79–1.34)
Tropical Latin America	35,235 (33,784–36,887)	193,001 (179,989–204,918)	36.45 (34.71–38.17)	73.91 (68.89–78.48)	2.28 (2.17–2.4)	8102 (7660–8506)	29,415 (26,981–31,339)	9.55 (8.91–10.06)	11.58 (10.59–12.34)	0.72 (0.66–0.78)	218,357 (209,209–228,658)	744,957 (694,956–786,849)	224.28 (213.27–235.19)	286.22 (266.67–302.45)	0.77 (0.68–0.87)
Western Europe	1,199,207 (1,139,433–1,245,117)	2,157,665 (1,991,049–2,289,198)	209.89 (200.26–217.33)	245.53 (229.06–259.17)	0.58 (0.39–0.78)	136,377 (127,108–142,174)	156,637 (136,857–169,939)	22.98 (21.45–23.93)	15.11 (13.53–16.25)	−1.14 (−1.22 to −1.06)	2,815,472 (2,680,130–2,917,395)	2,912,355 (2,641,021–3,112,519)	498.57 (476.35–516.24)	326.82 (301.67–347.23)	−1.41 (−1.46 to −1.36)
Western Sub-Saharan Africa	12,431 (10,672–14,473)	36,372 (29,707–43,211)	13.58 (11.77–15.7)	17.33 (14.51–20.23)	0.94 (0.84–1.04)	4162 (3557–4867)	10,312 (8668–12,100)	5.22 (4.48–6.04)	5.98 (5.12–6.88)	0.49 (0.43–0.54)	110,381 (93,741–130,867)	276,692 (224,568–328,426)	120.11 (102.58–141.26)	132.05 (110.39–155.48)	0.46 (0.4–0.52)

ASR = age-standardized rate, CI = confidence interval, DALY = disability-adjusted life year, EAPC = estimated annual percentage change, GBD = Global Burden of Disease, SDI = socio-demographic index, UI = uncertainty interval.

Furthermore, among the 5 SDI regions in 2021, the high SDI region had the highest ASPR, while the high–middle SDI region had the highest ASDR and ASDALYR. Among the 21 GBD regions in 2021, high-income Asia Pacific had the highest ASPR (282.83 per 100,000 [95% UI: 258.21–298.68]). From 1990 to 2021, the most significant increase in ASPR was observed in East Asia (EAPC = 3.31 [95% CI: 3.02–3.24]). In 2021, Australasia had the highest ASDR (14.62 per 100,000 [95% UI: 12.77–16.52]), and Central Europe had the highest ASDALYR (506.48 per 100,000 [95% UI: 467.97–544.57]). Additionally, from 1990 to 2021, the most significant decreases in ASDR (EAPC = −1.67 [95% CI: −1.76 to −1.57]) and ASDALYR (EAPC = −2.06 [95% CI: −2.17 to −1.96]) were also observed in Australasia (Table 3). Globally, the ASPR of CRC showed an overall increasing trend in most countries (Fig. 3G). Increasing ASDR and ASDALYR were predominantly concentrated in low-SDI countries in Africa and Southeast Asia (Fig. 3H–I). Notably, the ASPR and ASDALYR of CRC significantly increased with higher SDI levels, while ASDR peaked at an SDI range of 0.75 to 0.8 (Fig. 2J–L, Supplementary Material 1G–I, Supplemental Digital Content 1).

The BAPC model predicted that during the period 2021 to 2050, the number of prevalent cases of CRC would increase from 10.20 million cases in 2021 to 11.24 million cases in 2050, a 10.20% rise; the number of deaths would decrease from 0.94 million cases to 0.78 million cases, a 17.02% decrement; and DALYs would decline from 21.41 million cases to 18.81 million cases, a 12.14% decline. In contrast, the ASPR would decrease from 133.60 per 100,000 in 2021 to 121.38 per 100,000 in 2050, a 9.15% reduction; the ASDR would decline from 12.25 per 100,000 to 8.41 per 100,000, a 31.35% decrease; and the ASDALYR would drop from 280.33 per 100,000 to 203.11 per 100,000, a 27.55% decline. Consequently, for EC, SC, and CRC, the predicted results indicate that despite a decrease in ASR, the baseline burden of these 3 major GTCs remains substantial (Fig. 3M–O).

Globally, the 3rd-tier GBD risk factors affecting CRC deaths include diet high in processed meat (ASPAF: 5.48%), smoking (ASPAF: 4.45%), diet low in whole grains (ASPAF: 17.82%), diet low in fiber (ASPAF: 1.27%), diet low in milk (ASPAF: 15.07%), diet high in red meat (ASPAF: 14.64%), and diet low in calcium (ASPAF: 8.52%). Among these, the most notable were high-income North America (ASPAF: 11.52%) and Western Europe (ASPAF: 10.37%) for diet high in processed meat. Globally, dietary factors have the most significant impact on CRC, especially diet low in whole grains, which has the greatest impact on CRC. The trend of impact of 3rd-tier risk factors on CRC DALYs is consistent with that of deaths (Supplementary Material 2E–F, Supplemental Digital Content 2).

From 1990 to 2021, East Asia exhibited the largest overall increase in both CRC prevalent cases (2,680,460,706) and deaths (1,331,887,60.8). For prevalence, population growth contributed 2,576,014,383.3 (96.1%), epidemiological changes contributed 168,281,271.2 (6.28%), and population aging contributed 74,004,655.1 (2.76%); for deaths, population aging contributed 581,380,873.3 (43.65%), epidemiological changes contributed 560,788,665.16 (42.1%), and population growth contributed 189,718,222.27 (14.24%). South Asia had the 2nd largest increase in both prevalence (3,702,455,055.6) and deaths (614,724,664.41). For prevalence, epidemiological changes contributed 2,030,342,04.5 (54.84%), population growth contributed 1,039,145,577.9 (28.07%), and population aging contributed 632,962,733.2 (17.1%); for deaths, epidemiological changes contributed 294,372,223.12 (47.89%), population growth contributed 192,907,056.62 (31.38%), and population aging contributed 127,444,886.67 (20.73%). The decomposition trend of DALYs is similar to that of deaths (Supplementary Material 3G–I, Supplemental Digital Content 3).

## 4. Discussion

This study elucidates the temporal trends in disease burden of the 3 major GTCs from 1990 to 2021, along with their projections from 2022 to 2050. The key findings are as follows. First, over the past 3 decades, with the exception of DALYs for SC, the case numbers of disease burden for the remaining major GTCs have shown an increasing trend. However, with the exception of ASPR for CRC, the ASR for all 3 GTCs has exhibited a decreasing trend compared to 1990. Although the number of cases increased, the ASR actually rose due to factors such as population growth rate, aging population, and demographic changes. Second, between 1990 and 2021, the burden of the 3 major GTCs was most pronounced in the age group older than 50 years, highlighting the crucial role of population aging in the changes of GTC burden. Third, the regions with a heavier burden of the 3 major GTCs were predominantly concentrated in middle and high–middle SDI regions. Fourth, by 2050, the case numbers of disease burden for the 3 major GTCs will increase to varying degrees, yet the ASR burden will continue to show a decreasing trend. Fifth, the divergence between increasing case numbers and declining ASRs was clarified by our decomposition analysis. Population aging and growth were the predominant drivers of rising absolute cases, while epidemiological changes contributed variably across regions. For instance, population aging accounted for 50.87% of EC prevalent case increases in East Asia, whereas epidemiological changes contributed negatively to SC deaths (−7.58%) in the same region. These findings underscore that declining ASRs reflect demographic transitions rather than reduced absolute burden, and policy prioritization should distinguish between immutable demographic trends and modifiable epidemiological targets. It is worth noting that our findings sit at the translation interface between GBD estimates and national policy decisions, rather than seeking etiological explanations. Therefore, in the following discussion, we will examine the causes of the 3 primary GTCs burdens and provide corresponding recommendations.

This study demonstrates that East Asia exhibited the highest EC burden in 2021, while the most significant increase in EC burden from 1990 to 2021 was concentrated in high SDI regions. Additionally, the primary affected age group was 50 years and older. These results are consistent with previously summarized global epidemiological trends of EC.^[23]^ This may be related to high SDI regions, where esophageal adenocarcinoma constitutes the primary subtype, being influenced by intergenerational changes in obesity prevalence, resulting in an elevated risk of esophageal adenocarcinoma.^[23,24]^ Conversely, in East Asia, where China constitutes the predominant population, smoking and alcohol consumption represent the primary risk factors for increased esophageal squamous cell carcinoma burden in this region.^[23,24]^ Therefore, public health education on EC risk factors and the strengthening of EC screening are imperative. Since 1972, China has implemented large-scale screening programs for upper gastrointestinal cancers in 4 provinces, which have effectively reduced death rates in these regions.^[25,26]^ This may also account for the negative EAPC observed in East Asia. Additionally, another study based on the GLOBOCAN dataset projected the EC burden for 2040. The results indicated an increase from 604,100 new EC cases and 544,100 deaths in 2020 to 957,000 new cases and 880,000 EC deaths by 2040.^[4]^ These findings exhibit a similarly pronounced upward trend to the projections of our study’s BAPC model through 2050, further validating the accuracy of the predicted trends. Thus, we advocate that high SDI regions should prioritize EC screening to mitigate the increasing burden of EC. Furthermore, although East Asia as a whole bears a relatively high EC burden, significant heterogeneity persists within the region.^[27]^ Countries such as Japan and South Korea have established relatively comprehensive esophageal cancer screening systems (including endoscopic screening programs), with relatively high screening coverage rates.^[28,29]^ In contrast, China has a vast territory, and significant disparities exist among different regions in terms of healthcare resource accessibility, coverage of screening programs, and public health infrastructure. The burden of esophageal cancer in some underdeveloped areas may be underestimated, and the effectiveness of screening programs varies considerably.^[30,31]^ Therefore, when formulating regional prevention and control strategies, this internal heterogeneity should be fully taken into account to avoid treating East Asia as a homogeneous entity. For low- and middle-income countries or regions, given their limited health resources, prioritizing the promotion of cost-effective screening methods (such as initial endoscopic screening combined with pathological confirmation) and strengthening the capacity building of primary healthcare institutions may represent a more pragmatic approach to resource allocation.^[32]^

As reported by Smyth et al, the hotspots of SC incidence and death rates are located in Asia and South America.^[33]^ This corresponds to the regions of high-income Asia Pacific, Andean Latin America, and East Asia in the present study, which are the 3 areas with prominent ASR for SC. This may be associated with the high prevalence of *H pylori* infection in regions such as China, South Korea, and Japan, as *H pylori* infection represents the strongest and most consistent predictor of SC.^[33,34]^ Despite the persistently high current burden of SC, the ASR in the 3 aforementioned SC hotspot regions exhibited a decreasing trend from 1990 to 2021. This is consistent with the SC trends summarized in previous studies based on the GLOBOCAN dataset. Specifically, the incidence and death rates of SC have been steadily declining in North America, Northern Europe, and Australia; similar decreasing trends have been observed in regions with historically high SC incidence, including China, South Korea, and Japan.^[35]^ Therefore, we advocate that health policymakers in regions with a high SC burden should prioritize the implementation of SC screening and preventive measures, such as *H pylori* screening and eradication. Furthermore, this study reveals that the age-specific patterns of ASPR, ASDR, and ASDALYR in SC are significantly pronounced, with more severe cumulative effects observed in the elderly population. Therefore, current SC health management policies should prioritize interventions for individuals aged 60 and above. In China, asymptomatic individuals who received *H pylori* treatment experienced a 43% reduction in SC risk over 25 years. Specifically, the Matsu Islands in Taiwan Province, China, launched a large-scale screening program (positive ^13^C-urea breath test) and *H pylori* eradication initiative in 2004. By 2018, the *H pylori* prevalence in this region had decreased to 15% from 64.2%.^[36]^ In the United States, screening and treatment initiatives targeting *H pylori* have also been implemented, which have similarly effectively reduced the risk of SC.^[34]^ Global implementation of population-based *H pylori* screening and treatment would reduce 8.74 million DALYs, further decreasing the number of DALYs and ASR.^[37]^ However, it should be noted that the PAF calculated based on the GBD database reflects population-level association estimates; caution should be exercised when interpreting its implications for causal relationships at the individual level.

Danpanichkul et al conducted an epidemiological investigation on the ASIR and ASDR of GCs, including esophageal, gastric, colorectal, liver, pancreatic, and biliary tract cancers, based on the GBD 2021 dataset. The results demonstrated that, with the exception of ASIR for CRC, the ASIR burden of other GCs exhibited a decreasing trend. Furthermore, the highest ASIR for CRC in 2021 was observed in high SDI regions.^[1]^ This is consistent with the trend observed in the ASPR of the present study. Both ASIR and ASPR exhibit the highest CRC burden and a consistent upward trend in high SDI regions. This may be associated with the risk factors for CRC. Firstly, inflammatory bowel disease has been confirmed to increase the risk of CRC.^[38]^ Furthermore, the burden of inflammatory bowel disease tends to be heavier in high SDI regions.^[39]^ Secondly, high red meat consumption levels are also strongly associated with an increased risk of CRC.^[38]^ High red meat consumption levels are more prevalent in high SDI regions.^[6]^ This undoubtedly underscores to health policymakers, including those in high SDI regions, that CRC not only has a large patient base but also a high number of new cases. The ASR of CRC is most severe among individuals aged 80 and above, warranting prioritized attention from health authorities. Thus, implementing measures to reduce the CRC burden is imperative. Accordingly, we advocate that health policymakers, including those in high SDI regions, should implement effective strategies to address the CRC burden. Consistent with the findings of the present study that the GTC burden is predominantly concentrated in individuals aged 50 years and older, the United States Preventive Services Task Force strongly recommends CRC screening for all adults aged 50 to 75 years.^[40]^ Moreover, regardless of the CRC screening modality used (fecal, blood, or radiological), such public health measures yield significant net benefits and can significantly reduce the CRC burden.^[41]^ However, consensus remains elusive regarding the optimal CRC screening modality.^[41,42]^ Accordingly, different regions should select appropriately based on local conditions.

Collectively, the decomposition analyses for EC, SC, and CRC reveal that the divergence between rising case numbers and declining ASRs is not a uniform phenomenon but varies substantially by cancer type, region, and metric. Population aging consistently emerged as a major contributor across all 3 cancers, particularly for death and DALY metrics, reflecting the global demographic transition. However, the relative contributions of population growth and epidemiological changes varied markedly: for CRC prevalence in East Asia, population growth contributed 96.1%, whereas for SC deaths in the same region, epidemiological changes exerted a negative effect (−7.58%). This heterogeneity highlights the necessity for region-specific and cancer-specific policy responses. Regions with dominant population growth effects may prioritize health system capacity building, whereas those with substantial epidemiological contributions may benefit from targeted interventions addressing modifiable risk factors. The decomposition framework thus provides a more nuanced foundation for policy prioritization than descriptive trend analysis alone.

This study represents the 1st epidemiological investigation focusing on the 3 major GTCs, highlighting the current trends in GTC disease burden. Additionally, we utilized data on the 3 major GTCs from 1990 to 2021 for the 1st time to project their burden trends up to 2050 based on the BAPC model. Finally, this research has certain limitations. Firstly, due to inherent limitations of GBD research, this study was unable to conduct effective statistical analyses of cost-effectiveness, health system carrying capacity, and potential overdiagnosis hazards. Secondly, variations in public health capacity across global regions may have led to the underestimation of disease burden in low SDI regions. Due to inherent limitations of GBD research, this study was unable to conduct effective statistical analyses of cost-effectiveness, health system carrying capacity, and potential overdiagnosis hazards. Thirdly, regarding the risk factor analysis, the PAFs presented in this study were derived from aggregate-level GBD estimates, which may be subject to ecological fallacy when inferring individual-level causal relationships. Specifically, the PAFs do not account for unmeasured confounding factors, latency periods between exposure and outcome, or potential cross-sectional bias inherent in the GBD modeling framework. Fourthly, the BAPC model extrapolates from historical data without accounting for dynamic factors such as future risk factor changes, screening adoption, or treatment advancements; thus, the 2050 predictions should be regarded as scenario-based forecasts rather than definitive conclusions. The 95% CIs primarily reflect statistical estimation uncertainty and may underestimate the true variability. Future research should integrate real-time surveillance data and health economic models to develop more adaptive prediction frameworks. Fifthly, although prior comparative studies have demonstrated the favorable predictive performance of the BAPC model relative to alternative approaches such as APC Poisson models and Joinpoint regression, we acknowledge that direct sensitivity analysis within our own dataset was not performed. Future studies incorporating multi-model comparisons are warranted to further validate these findings.

## 5. Conclusion

From 1990 to 2021, globally, while the number of cases exhibited a predominantly increasing trend, the ASR for GTCs showed a decreasing trend, with the exception of the ASPR for CRC. Projections through 2050 also indicate a continued decreasing trend in GTC ASR. However, the burden of GTC remains severe in regions such as East Asia and high-income Asia Pacific. Therefore, we advocate that the prevention of GTC remains paramount over treatment, and the widespread implementation of endoscopic, laboratory, and radiological screening in GTC-endemic regions should be a key priority for health policymakers. Priority should be given to the 70 to 80 age group, and specific screening methods such as endoscopy and laboratory tests should be incorporated into existing public health programs.

## Author contributions

**Conceptualization:** Rao Wang, Wei-Guang Liu.

**Data curation:** Rao Wang, Wei-Guang Liu.

**Formal analysis:** Rao Wang, Wei-Guang Liu.

**Investigation:** Rao Wang, Wei-Guang Liu.

**Methodology:** Rao Wang, Wei-Guang Liu.

**Project administration:** Rao Wang, Wei-Guang Liu.

**Resources:** Rao Wang, Wei-Guang Liu.

**Software:** Rao Wang, Wei-Guang Liu.

**Supervision:** Wei-Guang Liu.

**Validation:** Rao Wang, Wei-Guang Liu.

**Visualization:** Rao Wang, Wei-Guang Liu.

**Writing – original draft:** Rao Wang, Wei-Guang Liu.

**Writing – review & editing:** Rao Wang, Wei-Guang Liu.









## References

[1] Danpanichkul P Suparan K Tothanarungroj P. Epidemiology of gastrointestinal cancers: a systematic analysis from the Global Burden of Disease Study 2021. Gut. 2024;74:26–34. 39242191 10.1136/gutjnl-2024-333227

[2] Sung H Ferlay J Siegel RL. Global cancer statistics 2020: GLOBOCAN estimates of incidence and mortality worldwide for 36 cancers in 185 countries. CA Cancer J Clin. 2021;71:209–49. 33538338 10.3322/caac.21660

[3] Rodriguez GM DePuy D Aljehani M. Trends in epidemiology of esophageal cancer in the US, 1975−2018. JAMA Netw Open. 2023;6:e2329497. 37606926 10.1001/jamanetworkopen.2023.29497 PMC10445206

[4] Morgan E Soerjomataram I Rumgay H. The global landscape of esophageal squamous cell carcinoma and esophageal adenocarcinoma incidence and mortality in 2020 and projections to 2040: new estimates from GLOBOCAN 2020. Gastroenterology. 2022;163:649–58.e2. 35671803 10.1053/j.gastro.2022.05.054

[5] López MJ Carbajal J Alfaro AL. Characteristics of gastric cancer around the world. Crit Rev Oncol Hematol. 2023;181:103841. 36240980 10.1016/j.critrevonc.2022.103841

[6] Li N Lu B Luo C. Incidence, mortality, survival, risk factor and screening of colorectal cancer: a comparison among China, Europe, and northern America. Cancer Lett. 2021;522:255–68. 34563640 10.1016/j.canlet.2021.09.034

[7] GBD 2021 Diseases and Injuries Collaborators. Global incidence, prevalence, years lived with disability (YLDs), disability-adjusted life-years (DALYs), and healthy life expectancy (HALE) for 371 diseases and injuries in 204 countries and territories and 811 subnational locations, 1990–2021: a systematic analysis for the Global Burden of Disease Study 2021. Lancet. 2024;403:2133–61. 38642570 10.1016/S0140-6736(24)00757-8 PMC11122111

[8] Li T Zhang H Lian M. Global status and attributable risk factors of breast, cervical, ovarian, and uterine cancers from 1990 to 2021. J Hematol Oncol. 2025;18:5. 39794860 10.1186/s13045-025-01660-y PMC11721161

[9] Lv B Lan JX Si YF. Epidemiological trends of subarachnoid hemorrhage at global, regional, and national level: a trend analysis study from 1990 to 2021. Mil Med Res. 2024;11:46. 38992778 10.1186/s40779-024-00551-6 PMC11241879

[10] Wang F Ma B Ma Q Liu X. Global, regional, and national burden of inguinal, femoral, and abdominal hernias: a systematic analysis of prevalence, incidence, deaths, and DALYs with projections to 2030. Int J Surg. 2024;110:1951–67. 38265437 10.1097/JS9.0000000000001071 PMC11020045

[11] GBD 2021 Forecasting Collaborators. Burden of disease scenarios for 204 countries and territories, 2022–2050: a forecasting analysis for the Global Burden of Disease Study 2021. Lancet. 2024;403:2204–56. 38762325 10.1016/S0140-6736(24)00685-8 PMC11121021

[12] Riebler A Held L. Projecting the future burden of cancer: Bayesian age-period-cohort analysis with integrated nested Laplace approximations. Biom J. 2017;59:531–49. 28139001 10.1002/bimj.201500263

[13] Knorr-Held L Rainer E. Projections of lung cancer mortality in West Germany: a case study in Bayesian prediction. Biostatistics. 2001;2:109–29. 12933560 10.1093/biostatistics/2.1.109

[14] Havulinna AS. Bayesian age-period-cohort models with versatile interactions and long-term predictions: mortality and population in Finland 1878–2050. Stat Med. 2014;33:845–56. 24105769 10.1002/sim.5954

[15] Huang Q Zi H Luo L Li X Zhu C Zeng X. Secular trends of morbidity and mortality of prostate, bladder, and kidney cancers in China, 1990 to 2019 and their predictions to 2030. BMC Cancer. 2022;22:1164. 36368976 10.1186/s12885-022-10244-9 PMC9650664

[16] Liu YM Wang W Zhang X. The rising death burden of atrial fibrillation and flutter in low-income regions and younger populations. Front Epidemiol. 2023;3:1122790. 38455885 10.3389/fepid.2023.1122790 PMC10910937

[17] Chen J Li C Bu CLN. Global burden of non-communicable diseases attributable to kidney dysfunction with projection into 2040. Chin Med J (Engl). 2025;138:1334–44. 38809055 10.1097/CM9.0000000000003143 PMC12150917

[18] Liu Q Wang H Chen Z. Global, regional, and national epidemiology of nasopharyngeal carcinoma in middle-aged and elderly patients from 1990 to 2021. Ageing Res Rev. 2025;104:102613. 39626854 10.1016/j.arr.2024.102613

[19] Carstensen B. Age-period-cohort models for the Lexis diagram. Stat Med. 2007;26:3018–45. 17177166 10.1002/sim.2764

[20] GBD 2021 Risk Factors Collaborators. Global burden and strength of evidence for 88 risk factors in 204 countries and 811 subnational locations, 1990–2021: a systematic analysis for the Global Burden of Disease Study 2021. Lancet. 2024;403:2162–203. 38762324 10.1016/S0140-6736(24)00933-4 PMC11120204

[21] Das Gupta P. A general method of decomposing a difference between two rates into several components. Demography. 1978;15:99–112. 631402

[22] Li R Cheng X Yang Y. Global deaths associated with population aging – 1990–2019. China CDC Wkly. 2023;5:1150–4. 38152634 10.46234/ccdcw2023.216 PMC10750162

[23] Thrift AP. Global burden and epidemiology of Barrett oesophagus and oesophageal cancer. Nat Rev Gastroenterol Hepatol. 2021;18:432–43. 33603224 10.1038/s41575-021-00419-3

[24] Dong J Thrift AP. Alcohol, smoking and risk of oesophago-gastric cancer. Best Pract Res Clin Gastroenterol. 2017;31:509–17. 29195670 10.1016/j.bpg.2017.09.002

[25] Li P Qi X Bai R. The spatiotemporal associations between esophageal and gastric cancers provide evidence for its joint endoscopic screening in China: a population-based study. BMC Med. 2024;22:364. 39232729 10.1186/s12916-024-03594-7 PMC11375892

[26] Zhao YX Zhao HP Zhao MY. Latest insights into the global epidemiological features, screening, early diagnosis and prognosis prediction of esophageal squamous cell carcinoma. World J Gastroenterol. 2024;30:2638–56. 38855150 10.3748/wjg.v30.i20.2638 PMC11154680

[27] Huang J Koulaouzidis A Marlicz W. Global burden, risk factors, and trends of esophageal cancer: an analysis of cancer registries from 48 countries. Cancers (Basel). 2021;13:141. 33466239 10.3390/cancers13010141 PMC7795486

[28] Hamashima C. Update version of the Japanese Guidelines for gastric cancer screening. Jpn J Clin Oncol. 2018;48:673–83. 29889263 10.1093/jjco/hyy077

[29] Ryu JE Choi E Lee K. Trends in the performance of the Korean national cancer screening program for gastric cancer from 2007 to 2016. Cancer Res Treat. 2022;54:842–9. 34607395 10.4143/crt.2021.482 PMC9296921

[30] Zhu H Ma X Ye T. Esophageal cancer in China: practice and research in the new era. Int J Cancer. 2023;152:1741–51. 36151861 10.1002/ijc.34301

[31] Zeng H Chen W Zheng R. Changing cancer survival in China during 2003–15: a pooled analysis of 17 population-based cancer registries. Lancet Glob Health. 2018;6:e555–67. 29653628 10.1016/S2214-109X(18)30127-X

[32] Li H Ding C Zeng H. Improved esophageal squamous cell carcinoma screening effectiveness by risk-stratified endoscopic screening: evidence from high-risk areas in China. Cancer Commun (Lond). 2021;41:715–25. 34146456 10.1002/cac2.12186 PMC8360639

[33] Smyth EC Nilsson M Grabsch HI Van Grieken NC Lordick F. Gastric cancer. Lancet. 2020;396:635–48. 32861308 10.1016/S0140-6736(20)31288-5

[34] Moss SF Shah SC Tan MC El-Serag HB. Evolving concepts in *Helicobacter pylori* management. Gastroenterology. 2024;166:267–83. 37806461 10.1053/j.gastro.2023.09.047 PMC10843279

[35] Thrift AP El-Serag HB. Burden of gastric cancer. Clin Gastroenterol Hepatol. 2020;18:534–42. 31362118 10.1016/j.cgh.2019.07.045 PMC8859863

[36] Chiang TH Chang WJ Chen SLS. Mass eradication of *Helicobacter pylori* to reduce gastric cancer incidence and mortality: a long-term cohort study on Matsu Islands. Gut. 2021;70:243–50. 32792335 10.1136/gutjnl-2020-322200 PMC7815911

[37] Ford AC Yuan Y Moayyedi P. *Helicobacter pylori* eradication therapy to prevent gastric cancer: systematic review and meta-analysis. Gut. 2020;69:2113–21. 32205420 10.1136/gutjnl-2020-320839

[38] Pinheiro M Moreira DN Ghidini M. Colon and rectal cancer: an emergent public health problem. World J Gastroenterol. 2024;30:644–51. 38515957 10.3748/wjg.v30.i7.644 PMC10950624

[39] Lin D Jin Y Shao X. Global, regional, and national burden of inflammatory bowel disease, 1990–2021: insights from the global burden of disease 2021. Int J Colorectal Dis. 2024;39:139. 39243331 10.1007/s00384-024-04711-x PMC11380638

[40] Davidson KW Barry MJ Mangione CM. Screening for colorectal cancer: US preventive services task force recommendation statement. JAMA. 2021;325:1965–77. 34003218 10.1001/jama.2021.6238

[41] Metaxas G Papachristou A Stathaki M. Colorectal cancer screening: modalities and adherence. World J Gastroenterol. 2024;30:3048–51. 38983962 10.3748/wjg.v30.i24.3048 PMC11230065

[42] Lin JS Perdue LA Henrikson NB Bean SI Blasi PR. Screening for colorectal cancer: updated evidence report and systematic review for the US preventive services task force. JAMA. 2021;325:1978–98. 34003220 10.1001/jama.2021.4417 PMC13509038

