# Supplementary material for: Global burden of esophagus cancer, stomach cancer and colon and rectal cancer from 1990 to 2050: A cross-sectional study
Source: Medicine (Baltimore). 2026 Aug 21;105(34):e50376. doi: 10.1097/MD.0000000000050376 (PMC13502806; doi:10.1097/MD.0000000000050376)
Supplement: Supplementary file 1 [file medi-105-e50376-s004.docx]

| **Supplementary Table. Projected results of the BAPC model for Colon and rectal cancer up to 2050** | | | | | | |
| --- | --- | --- | --- | --- | --- | --- |
|  | **Prevalence Cases** | **ASPR** | **Deaths Cases** | **ASDR** | **DALYs Cases** | **ASDALYR** |
| **1990** | 5721620.704 (5721611.19126128, 5721630.21673872) | 108.315162 (108.315161999863, 108.315162000137) | 820082.5805 (820073.28204644, 820091.87895356) | 15.524863 (15.5248629998688, 15.5248630001312) | 18876967.95 (18876958.4251424, 18876977.4748576) | 357.3571104 (357.357110399863, 357.357110400137) |
| **1991** | 5864342.434 (5864333.01791392, 5864351.85008608) | 109.3007471 (109.300747099868, 109.300747100132) | 830704.5597 (830695.900522735, 830713.218877265) | 15.48283205 (15.4828320498873, 15.4828320501127) | 19098999.21 (19098989.7333806, 19099008.6866194) | 355.9708366 (355.970836599866, 355.970836600134) |
| **1992** | 6025898.593 (6025889.20564476, 6025907.98035524) | 110.6221712 (110.622171199871, 110.622171200129) | 842644.5403 (842635.941358315, 842653.139241685) | 15.46909015 (15.4690901498907, 15.4690901501093) | 19354874.95 (19354865.4987976, 19354884.4012024) | 355.3126985 (355.312698499869, 355.312698500131) |
| **1993** | 6199205.515 (6199196.14945634, 6199214.88054366) | 112.1426372 (112.142637199873, 112.142637200127) | 854585.7285 (854577.144330305, 854594.312669695) | 15.45931927 (15.4593192698925, 15.4593192701075) | 19662345.03 (19662335.602356, 19662354.457644) | 355.6886797 (355.688679699871, 355.688679700129) |
| **1994** | 6353414.848 (6353405.50322695, 6353424.19277305) | 113.2855091 (113.285509099876, 113.285509100124) | 863292.4526 (863283.884311408, 863301.020888592) | 15.39306457 (15.3930645698945, 15.3930645701055) | 19856355.75 (19856346.3456026, 19856365.1543974) | 354.0517068 (354.051706799874, 354.051706800126) |
| **1995** | 6514282.589 (6514273.26388556, 6514291.91411444) | 114.5144302 (114.514430199878, 114.514430200122) | 869313.1939 (869304.640889627, 869321.746910373) | 15.28163749 (15.2816374898964, 15.2816374901036) | 19957152.88 (19957143.4966689, 19957162.2633311) | 350.826351 (350.826350999876, 350.826351000124) |
| **1996** | 6654837.14 (6654827.83610152, 6654846.44389848) | 115.3467085 (115.34670849988, 115.34670850012) | 872458.5941 (872450.058348126, 872467.129851874) | 15.1221172 (15.1221171998982, 15.1221172001018) | 19970210.37 (19970201.0098168, 19970219.7301832) | 346.1389042 (346.138904199879, 346.138904200121) |
| **1997** | 6765077.167 (6765067.88581484, 6765086.44818515) | 115.6380926 (115.638092599882, 115.638092600118) | 875699.5504 (875691.029452301, 875708.071347699) | 14.96867267 (14.9686726699001, 14.9686726700999) | 19990144.91 (19990135.5747644, 19990154.2452356) | 341.6993142 (341.699314199881, 341.699314200119) |
| **1998** | 6899221.882 (6899212.62320278, 6899231.14079722) | 116.3399164 (116.339916399884, 116.339916400116) | 881704.0979 (881695.589313265, 881712.606486735) | 14.86796378 (14.8679637799017, 14.8679637800983) | 20083223.35 (20083214.0396252, 20083232.6603748) | 338.6585569 (338.658556899883, 338.658556900117) |
| **1999** | 7068343.175 (7068333.94044651, 7068352.40955349) | 117.616344 (117.616343999886, 117.616344000114) | 889152.3099 (889143.813919702, 889160.805880298) | 14.79538293 (14.7953829299033, 14.7953829300967) | 20270300.46 (20270291.1768755, 20270309.7431245) | 337.2952577 (337.295257699885, 337.295257700115) |
| **2000** | 7255969.386 (7255960.1747672, 7255978.5972328) | 119.1613175 (119.161317499888, 119.161317500112) | 895833.6711 (895825.187864513, 895842.154335487) | 14.71184824 (14.7118482399049, 14.7118482400951) | 20421334.67 (20421325.4137889, 20421343.9262111) | 335.3698195 (335.369819499887, 335.369819500113) |
| **2001** | 7442036.085 (7442026.89972762, 7442045.27027238) | 120.6290915 (120.62909149989, 120.62909150011) | 901396.0113 (901387.54331319, 901404.47928681) | 14.61086464 (14.6108646399065, 14.6108646400935) | 20513453.46 (20513444.2324987, 20513462.6875013) | 332.5056779 (332.505677899889, 332.505677900111) |
| **2002** | 7597315.686 (7597306.52905076, 7597324.84294924) | 121.5523309 (121.552330899892, 121.552330900108) | 906874.8305 (906866.379222673, 906883.281777327) | 14.50943386 (14.509433859908, 14.509433860092) | 20612344.38 (20612335.1832986, 20612353.5767014) | 329.784704 (329.784703999891, 329.784704000109) |
| **2003** | 7756891.699 (7756882.57031262, 7756900.82768738) | 122.5149731 (122.514973099894, 122.514973100106) | 913081.2864 (913072.848727893, 913089.724072107) | 14.4215149 (14.4215148999095, 14.4215149000905) | 20748380.86 (20748371.6931073, 20748390.0268927) | 327.7069502 (327.706950199893, 327.706950200107) |
| **2004** | 7912492.197 (7912483.09924217, 7912501.29475783) | 123.3832396 (123.383239599896, 123.383239600104) | 914697.5038 (914689.084917506, 914705.922682494) | 14.26331154 (14.2633115399111, 14.2633115400889) | 20765927.39 (20765918.2559099, 20765936.5240901) | 323.8129441 (323.812944099895, 323.812944100105) |
| **2005** | 8126522.051 (8126512.98413126, 8126531.11786874) | 125.1225868 (125.122586799898, 125.122586800102) | 915532.3077 (915523.908682207, 915540.706717793) | 14.09628497 (14.0962849699127, 14.0962849700873) | 20806536.91 (20806527.8082841, 20806546.0117159) | 320.3544769 (320.354476899897, 320.354476900103) |
| **2006** | 8285259.346 (8285250.31334411, 8285268.37865589) | 125.9424389 (125.9424388999, 125.9424389001) | 910183.8492 (910175.478314915, 910192.220085085) | 13.83550823 (13.8355082299145, 13.8355082300855) | 20611876.3 (20611867.2331765, 20611885.3668235) | 313.3166825 (313.316682499899, 313.316682500101) |
| **2007** | 8464321.708 (8464312.71066889, 8464330.70533111) | 126.9940934 (126.994093399902, 126.994093400098) | 911152.9687 (911144.621620989, 911161.315779011) | 13.6704451 (13.670445099916, 13.670445100084) | 20635831.27 (20635822.2387702, 20635840.3012298) | 309.6088232 (309.608823199902, 309.608823200098) |
| **2008** | 8655988.553 (8655979.59012712, 8655997.51587288) | 128.2148941 (128.214894099904, 128.214894100096) | 917625.0747 (917616.748402185, 917633.400997815) | 13.59211615 (13.5921161499177, 13.5921161500823) | 20822112.28 (20822103.283796, 20822121.276204) | 308.4228802 (308.422880199903, 308.422880200097) |
| **2009** | 8783652.906 (8783643.97879892, 8783661.83320108) | 128.5069255 (128.506925499906, 128.506925500094) | 920135.7425 (920127.439084063, 920144.045915937) | 13.46180417 (13.4618041699191, 13.4618041700809) | 20847544.63 (20847535.6706405, 20847553.5893595) | 305.0045229 (305.004522899906, 305.004522900094) |
| **2010** | 8951990.397 (8951981.50374776, 8951999.29025224) | 129.3828872 (129.382887199908, 129.382887200092) | 925705.8991 (925697.615512511, 925714.182687489) | 13.37920358 (13.3792035799206, 13.3792035800794) | 21007150.66 (21007141.7362451, 21007159.5837549) | 303.6158087 (303.615808699907, 303.615808700093) |
| **2011** | 9121732.431 (9121723.57368649, 9121741.28831351) | 130.2459382 (130.24593819991, 130.24593820009) | 929713.7632 (929705.502820493, 929722.023579507) | 13.2750486 (13.2750485999219, 13.2750486000781) | 21100922.21 (21100913.3227672, 21100931.0972328) | 301.2924827 (301.292482699909, 301.292482700091) |
| **2012** | 9257164.879 (9257156.05952008, 9257173.69847992) | 130.5960803 (130.596080299912, 130.596080300088) | 931667.8146 (931659.581228774, 931676.047971226) | 13.14356677 (13.1435667699234, 13.1435667700766) | 21145009.31 (21145000.4608644, 21145018.1591356) | 298.3046505 (298.304650499911, 298.304650500089) |
| **2013** | 9386209.084 (9386200.30140778, 9386217.86659222) | 130.8482453 (130.848245299913, 130.848245300087) | 932986.9478 (932978.739199114, 932995.156400887) | 13.00628442 (13.0062844199249, 13.0062844200751) | 21168979.35 (21168970.537945, 21168988.162055) | 295.1057 (295.105699999913, 295.105700000087) |
| **2014** | 9514450.3 (9514441.55378697, 9514459.04621303) | 131.1002081 (131.100208099915, 131.100208100085) | 935074.9784 (935066.79304963, 935083.16375037) | 12.88445685 (12.8844568499264, 12.8844568500736) | 21200447.94 (21200439.1641146, 21200456.7158854) | 292.1223034 (292.122303399914, 292.122303400086) |
| **2015** | 9689243.025 (9689234.31209869, 9689251.73790131) | 131.9904405 (131.990440499917, 131.990440500083) | 942561.1672 (942553.003481769, 942569.330918231) | 12.83991571 (12.8399157099275, 12.8399157100725) | 21408413.64 (21408404.8981864, 21408422.3818136) | 291.633303 (291.633302999916, 291.633303000084) |
| **2016** | 9858219.585 (9858210.90727701, 9858228.26272299) | 132.7720771 (132.772077099918, 132.772077100082) | 950362.2823 (950354.139620468, 950370.424979532) | 12.79963113 (12.7996311299288, 12.7996311300712) | 21626497.47 (21626488.7631387, 21626506.1768613) | 291.2691248 (291.269124799918, 291.269124800082) |
| **2017** | 10009839.16 (10009830.5181737, 10009847.8018263) | 133.307465 (133.30746499992, 133.30746500008) | 954002.3365 (953994.21634584, 954010.45665416) | 12.70506259 (12.7050625899302, 12.7050625900698) | 21717371.16 (21717362.4882589, 21717379.8317411) | 289.2241973 (289.224197299919, 289.224197300081) |
| **2018** | 10193672.19 (10193663.5830214, 10193680.7969786) | 134.2949599 (134.294959899921, 134.294959900079) | 959973.5986 (959965.496651666, 959981.700548334) | 12.64702391 (12.6470239099312, 12.6470239100688) | 21900003.4 (21899994.762565, 21900012.037435) | 288.5182125 (288.518212499921, 288.518212500079) |
| **2019** | 10357207.73 (10357199.1574485, 10357216.3025515) | 135.0391816 (135.039181599923, 135.039181600077) | 964707.9394 (964699.85805197, 964716.02074803) | 12.57803977 (12.5780397699324, 12.5780397700676) | 22052068.47 (22052059.8659854, 22052077.0740146) | 287.5189297 (287.518929699922, 287.518929700078) |
| **2020** | 10092517.09 (10092508.6065764, 10092525.5734236) | 133.5747678 (133.574767799923, 133.574767800077) | 935755.2344 (935747.211693798, 935763.257106202) | 12.38474872 (12.3847487199325, 12.3847487200675) | 21337472.93 (21337464.416001, 21337481.443999) | 282.4020972 (282.402097199923, 282.402097200077) |
| **2021** | 10202779.13 (10202770.6574573, 10202787.6025427) | 133.599692 (133.599691999925, 133.599692000075) | 935482.8504 (935474.532039216, 935491.168760784) | 12.24962523 (12.2496252299275, 12.2496252300725) | 21408316.99 (21408308.4978968, 21408325.4821032) | 280.3299492 (280.329949199924, 280.329949200076) |
| **2022** | 10405210.7 (10405138.7220721, 10405282.6779279) | 134.8359574 (134.83595739907, 134.83595740093) | 936346.3952 (936328.159182833, 936364.631217167) | 12.13364787 (12.1336478697772, 12.1336478702228) | 21762656.21 (21762545.1646729, 21762767.2553271) | 282.0114527 (282.011452698563, 282.011452701437) |
| **2023** | 10482402.28 (10482299.4460921, 10482505.1139079) | 134.4779656 (134.47796559868, 134.47796560132) | 934334.4707 (934306.439715487, 934362.501684513) | 11.986508 (11.9865079996492, 11.9865080003508) | 21778561.66 (21778402.6577617, 21778720.6622383) | 279.395561 (279.395560997963, 279.395561002037) |
| **2024** | 10557646.95 (10557503.7850185, 10557790.1149815) | 134.123017 (134.123016998184, 134.123017001816) | 932066.4433 (932026.086276619, 932106.800323381) | 11.8408547 (11.8408546994925, 11.8408547005075) | 21785316.05 (21785094.5724856, 21785537.5275144) | 276.7579112 (276.757911197187, 276.757911202814) |
| **2025** | 10631991.7 (10631801.0327871, 10632182.3672129) | 133.7863241 (133.786324097602, 133.786324102398) | 929498.8619 (929444.300658369, 929553.423141631) | 11.69623148 (11.6962314793169, 11.6962314806831) | 21781146.31 (21780851.5456177, 21781441.0743823) | 274.0803023 (274.080302296292, 274.080302303708) |
| **2026** | 10703201.22 (10702956.9299097, 10703445.5100903) | 133.442006 (133.442005996957, 133.442006003043) | 926587.3243 (926516.93881741, 926657.70978259) | 11.55221403 (11.5522140291265, 11.5522140308735) | 21767510.83 (21767133.8668382, 21767887.7931618) | 271.3861257 (271.386125695295, 271.386125704705) |
| **2027** | 10770366.17 (10770062.6590642, 10770669.6809358) | 133.0796732 (133.079673196249, 133.079673203751) | 923408.4423 (923320.727393266, 923496.157206734) | 11.40972292 (11.409722918919, 11.409722921081) | 21748253.66 (21747786.6507084, 21748720.6692916) | 268.7234997 (268.723499694242, 268.723499705758) |
| **2028** | 10834393.01 (10834025.584926, 10834760.435074) | 132.7106035 (132.710603495498, 132.710603504502) | 920111.7671 (920005.487124346, 920218.047075654) | 11.27045953 (11.2704595287005, 11.2704595312995) | 21723583.77 (21723020.0189781, 21724147.5210219) | 266.0924253 (266.092425293103, 266.092425306897) |
| **2029** | 10895908.18 (10895472.6332532, 10896343.7267468) | 132.3440981 (132.344098094709, 132.344098105291) | 916608.1997 (916482.28548494, 916734.11391506) | 11.13332487 (11.1333248684728, 11.1333248715272) | 21689797.46 (21689131.0370518, 21690463.8829482) | 263.4490523 (263.44905229192, 263.44905230808) |
| **2030** | 10954391.86 (10953884.2165621, 10954899.5034379) | 131.9756195 (131.9756194939, 131.9756195061) | 912878.6628 (912732.129265686, 913025.196334314) | 10.99812099 (10.9981209882358, 10.9981209917642) | 21643489.3 (21642714.7884495, 21644263.8115505) | 260.755042 (260.755041990689, 260.755042009311) |
| **2031** | 11007828.99 (11007245.3192023, 11008412.6607977) | 131.5812566 (131.581256593029, 131.581256606971) | 908799.4743 (908631.360924842, 908967.587675158) | 10.86326622 (10.8632662179913, 10.8632662220087) | 21585212.24 (21584324.4998739, 21586099.9801261) | 258.0172125 (258.017212489369, 258.017212510631) |
| **2032** | 11057131.77 (11056468.0875675, 11057795.4524325) | 131.1722166 (131.172216592102, 131.172216607898) | 904343.7205 (904153.063342495, 904534.377657505) | 10.72834916 (10.7283491577328, 10.7283491622672) | 21519796.15 (21518790.1662501, 21520802.1337499) | 255.2921879 (255.292187888085, 255.292187911915) |
| **2033** | 11104605.73 (11103858.4690285, 11105352.9909715) | 130.7757594 (130.775759391177, 130.775759408823) | 899702.1132 (899488.08112626, 899916.14527374) | 10.59553396 (10.595533957479, 10.595533962521) | 21449673.76 (21448545.0105273, 21450802.5094727) | 252.606661 (252.606660986712, 252.606661013288) |
| **2034** | 11149660.32 (11148826.2068672, 11150494.4331328) | 130.3842137 (130.384213690256, 130.384213709744) | 894851.7924 (894613.654859324, 895089.929940676) | 10.46440376 (10.4644037572236, 10.4644037627764) | 21371190.27 (21369934.7140538, 21372445.8259462) | 249.9148638 (249.91486378533, 249.91486381467) |
| **2035** | 11189781.37 (11188857.3419411, 11190705.3980589) | 129.9680426 (129.968042589277, 129.968042610723) | 889808.5012 (889545.576034706, 890071.426365294) | 10.33502491 (10.3350249069456, 10.3350249130544) | 21279516.97 (21278130.9874556, 21280902.9525444) | 247.1591782 (247.159178183897, 247.159178216103) |
| **2036** | 11223359.35 (11222342.3829118, 11224376.3170882) | 129.5090443 (129.50904428824, 129.50904431176) | 884442.9129 (884154.539257467, 884731.286542532) | 10.2057996 (10.2057995966654, 10.2057996033346) | 21174253.26 (21172733.4305342, 21175773.0894658) | 244.3348037 (244.334803682451, 244.334803717549) |
| **2037** | 11253221.78 (11252108.666892, 11254334.893108) | 129.0403206 (129.040320587263, 129.040320612737) | 878659.7959 (878345.317665657, 878974.274134343) | 10.07556271 (10.0755627064035, 10.0755627135965) | 21060938.92 (21059281.7749039, 21062596.0650961) | 241.5050874 (241.505087380995, 241.505087419005) |
| **2038** | 11282876.5 (11281664.227717, 11284088.772283) | 128.60162 (128.601619986171, 128.601620013829) | 872628.5388 (872287.384902745, 872969.692697254) | 9.946173193 (9.94617318911838, 9.94617319688162) | 20944118.21 (20942320.5214235, 20945915.8985765) | 238.7199339 (238.719933879528, 238.719933920472) |
| **2039** | 11309621.95 (11308307.8535181, 11310936.0464819) | 128.1613691 (128.16136908508, 128.16136911492) | 866313.9451 (865945.63037172, 866682.25982828) | 9.817125782 (9.8171257778305, 9.8171257861695) | 20819831.78 (20817890.7887517, 20821772.7712483) | 235.9316833 (235.931683278007, 235.931683321993) |
| **2040** | 11328386.93 (11326968.7491328, 11329805.1108672) | 127.6619744 (127.661974384044, 127.661974415956) | 859740.0339 (859344.11394624, 860135.95385376) | 9.688591224 (9.68859121953979, 9.68859122846021) | 20681825.14 (20679738.6216548, 20683911.6583452) | 233.0678363 (233.067836276468, 233.067836323532) |
| **2041** | 11338372.4 (11336847.943444, 11339896.856556) | 127.0955425 (127.095542482887, 127.095542517113) | 852892.4685 (852468.509742614, 853316.427257386) | 9.56035198 (9.56035197524603, 9.56035198475398) | 20529280.14 (20527046.1038112, 20531514.1761888) | 230.119448 (230.119447974953, 230.119448025047) |
| **2042** | 11344839.75 (11343206.4483863, 11346473.0516137) | 126.5216218 (126.521621781786, 126.521621818214) | 845730.7688 (845278.34179053, 846183.19580947) | 9.431885409 (9.43188540394887, 9.43188541405113) | 20368535.86 (20366152.1284472, 20370919.5915528) | 227.1570377 (227.157037673422, 227.157037726578) |
| **2043** | 11353048.93 (11351304.1367374, 11354793.7232626) | 125.9989189 (125.998918880629, 125.998918919371) | 838402.9716 (837921.696770736, 838884.246429264) | 9.304801622 (9.30480161664857, 9.30480162735143) | 20206211.94 (20203676.3098329, 20208747.5701671) | 224.2534914 (224.253491371876, 224.253491428124) |
| **2044** | 11358375.44 (11356517.0184791, 11360233.8615209) | 125.4759454 (125.475945379471, 125.475945420529) | 830810.4215 (830300.01189616, 831320.83110384) | 9.177960669 (9.17796066336614, 9.17796067463387) | 20038068.58 (20035379.3622349, 20040757.7977651) | 221.360494 (221.360493970314, 221.360494029686) |
| **2045** | 11353017.23 (11351043.801196, 11354990.658804) | 124.866671 (124.866670978303, 124.866671021697) | 822894.1623 (822354.393632323, 823433.930967677) | 9.050638479 (9.05063847305822, 9.05063848494178) | 19856366.49 (19853522.7454989, 19859210.2345011) | 218.3911405 (218.391140468727, 218.391140531273) |
| **2046** | 11336342.79 (11334253.1130416, 11338432.4669584) | 124.1651979 (124.165197877122, 124.165197922878) | 814690.2841 (814120.938055117, 815259.630144883) | 8.923175868 (8.9231758617678, 8.9231758742322) | 19659490.88 (19656492.0061318, 19662489.7538682) | 215.3273435 (215.327343467156, 215.327343532844) |
| **2047** | 11314985.53 (11312777.78613, 11317193.27387) | 123.4446028 (123.444602775935, 123.444602824065) | 806207.7797 (805608.639693582, 806806.919706418) | 8.795592253 (8.79559224647315, 8.79559225952685) | 19453534.07 (19450379.1948681, 19456688.9451319) | 212.2348083 (212.23480826556, 212.23480833444) |
| **2048** | 11296362.35 (11294034.270111, 11298690.429889) | 122.7855575 (122.785557474691, 122.785557525309) | 797575.0391 (796945.915305273, 798204.162894727) | 8.66922402 (8.66922401315261, 8.66922402684739) | 19246780.38 (19243468.3047, 19250092.4553) | 209.2024481 (209.202448063991, 209.202448136009) |
| **2049** | 11275211.24 (11272761.2194291, 11277661.2605709) | 122.1305927 (122.130592673441, 122.130592726559) | 788671.8331 (788012.639993534, 789331.026206466) | 8.542718742 (8.54271873484923, 8.54271874915077) | 19035506.87 (19032036.9394863, 19038976.8005137) | 206.1883973 (206.188397262399, 206.188397337601) |
| **2050** | 11242285.54 (11239713.1065351, 11244857.9734649) | 121.3797882 (121.379788172233, 121.379788227767) | 779403.0572 (778713.795903909, 780092.318496091) | 8.414995122 (8.41499511456262, 8.41499512943738) | 18811754.04 (18808126.5527411, 18815381.5272589) | 203.105206 (203.10520596082, 203.10520603918) |
